# Vegan diet and nutritional status in infants, children and adolescents: A position paper based on a systematic search by the ESPGHAN Nutrition Committee

**DOI:** 10.1002/jpn3.70182

**Published:** 2025-08-17

**Authors:** Elvira Verduci, Jutta Kӧglmeier, Nadja Haiden, Laura Kivelä, Barbara de Koning, Susan Hill, Veronica Luque, Sissel J. Moltu, Lorenzo Norsa, Miguel Saenz De Pipaon, Francesco Savino, Jiri Bronsky

**Affiliations:** ^1^ Metabolic Disease Unit, Department of Pediatrics, Vittore Buzzi Children's Hospital University of Milan Milan Italy; ^2^ Departiment of Health Science University of Milan Milan Italy; ^3^ Department of Paediatric Gastroenterology Great Ormond Street Hospital for Children NHS Foundation Trust London London UK; ^4^ Department of Neonatology Kepler University Hospital Linz Austria; ^5^ Celiac Disease Research Center, Faculty of Medicine and Health Technology Tampere University Tampere Finland; ^6^ Department of Pediatrics Tampere University Hospital, Wellbeing Services County of Pirkanmaa Tampere Finland; ^7^ Children's Hospital Helsinki University Hospital Helsinki Finland; ^8^ Department of Pediatric Research University of Oslo Oslo Norway; ^9^ Paediatric Gastroenterology Erasmus MC‐Sophia Children's Hospital Rotterdam the Netherlands; ^10^ Serra Hunter Fellow, Pediatric Nutrition Research Unit Universitat Rovira i Virgili‐IISPV Tarragona Spain; ^11^ Department of Neonatal Intensive Care Oslo University Hospital Oslo Norway; ^12^ Department of Pediatrics, Vittore Buzzi Children's Hospital University of Milan Milan Italy; ^13^ Neonatology Hospital La Paz Institute for Health Research IdiPAZ, Universidad Autónoma de Madrid Madrid Spain; ^14^ Department of Pediatrics, Regina Margherita Children Hospital University of Turin Turin Italy; ^15^ Department of Paediatrics University Hospital Motol Prague Czech Republic

**Keywords:** growth, micronutrients, nutritional intake, plant‐based diet, vitamin B12

## Abstract

Vegan and other plant‐based diets are becoming increasingly popular in the paediatric age group. There is limited evidence in the current medical literature to determine whether a vegan diet is adequate for children, since the currently available society position papers are based on narrative reviews and expert opinion. Updated evidence‐based recommendations are needed to guide clinical practice. This position paper presents findings from a literature review performed using a systematic search strategy, following the Preferred Reporting Items for Systematic Reviews and Meta‐Analysis guidelines. We analyzed the current evidence on the effect of vegan diet compared to omnivorous diet on body growth, nutritional adequacy and laboratory biomarkers in infants, children and adolescents. Observational studies, cohort studies and clinical trials published over the last 15 years in MEDLINE/PubMed, EMBASE and Cochrane Library were retrieved. Our position paper aims to update the evidence for or against the adequacy of a vegan diet in infants, children and adolescents and to provide evidence‐based recommendations. A total of 10 articles were accepted and included in the final review, providing information on approximately 1500 children following a vegan diet. Several articles assessed more than one outcome: seven addressed body growth, five evaluated nutritional adequacy and five examined laboratory biomarkers. To complement the primary data, three systematic reviews and meta‐analyses were also included. Current evidence is inconclusive to determine whether a strictly vegan diet supports normal childhood growth, although no significant differences in height or body mass index *z*‐scores were observed compared to omnivorous peers. We recommend that dietary intake, growth and nutritional status should be regularly monitored in vegan children. Focusing on dietary intakes (e.g., protein, omega‐3, calcium and iron) and ensuring supplementation with specific micronutrients, including vitamin B12, is essential during paediatric age when following a strict vegan diet. Clinical research, well‐designed prospective studies and high‐quality trials are required to address current research gaps.

## Abstract (highlights)

**What is Known:**

- Growing awareness of the environmental benefits of avoiding meat and animal products has led to a significant increase in people adopting a vegan lifestyle.
- Current statements and position papers from paediatric societies regarding a vegan diet in children are based on expert opinions and narrative literature reviews, which are inconsistent and reflect differing viewpoints.
- There is limited evidence to establish whether a vegan diet can safely support healthy growth and nutritional adequacy in children.

**What is New:**

- There are no proven health benefits of a vegan diet in childhood.
- There is inconclusive evidence for adequate growth in children adopting a vegan diet.
- We recommend that infants, children and adolescents on a vegan diet should receive regular counselling from an appropriately qualified dietitian, be monitored by a paediatrician and have specific micronutrient supplements, including vitamin B12.
- Formal guidance is needed for healthcare professionals on how to support families and to evaluate and monitor a vegan diet in infants, children and adolescents.

## Graphical abstract

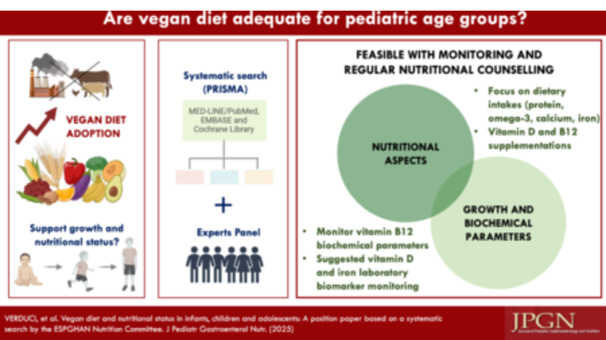

## 1 INTRODUCTION

Exclusively plant‐based diets (PBDs) have become increasingly popular,^1^ and many parents adopting PBD are choosing the same dietary pattern for their children. Whilst some people are concerned about animal welfare, the carbon footprint of greenhouse gas emissions by livestock, and/or loss of rainforest for animal grazing, others are vegan for cultural and religious reasons.^2^, ^3^, ^4^, ^5^ There is a growing perception that avoiding animal products may have health benefits in adults and that a PBD diet might be superior to an omnivorous diet, that is, a diet that includes both plant‐based and animal‐based foods.^6^, ^7^, ^8^

**Figure 1:**
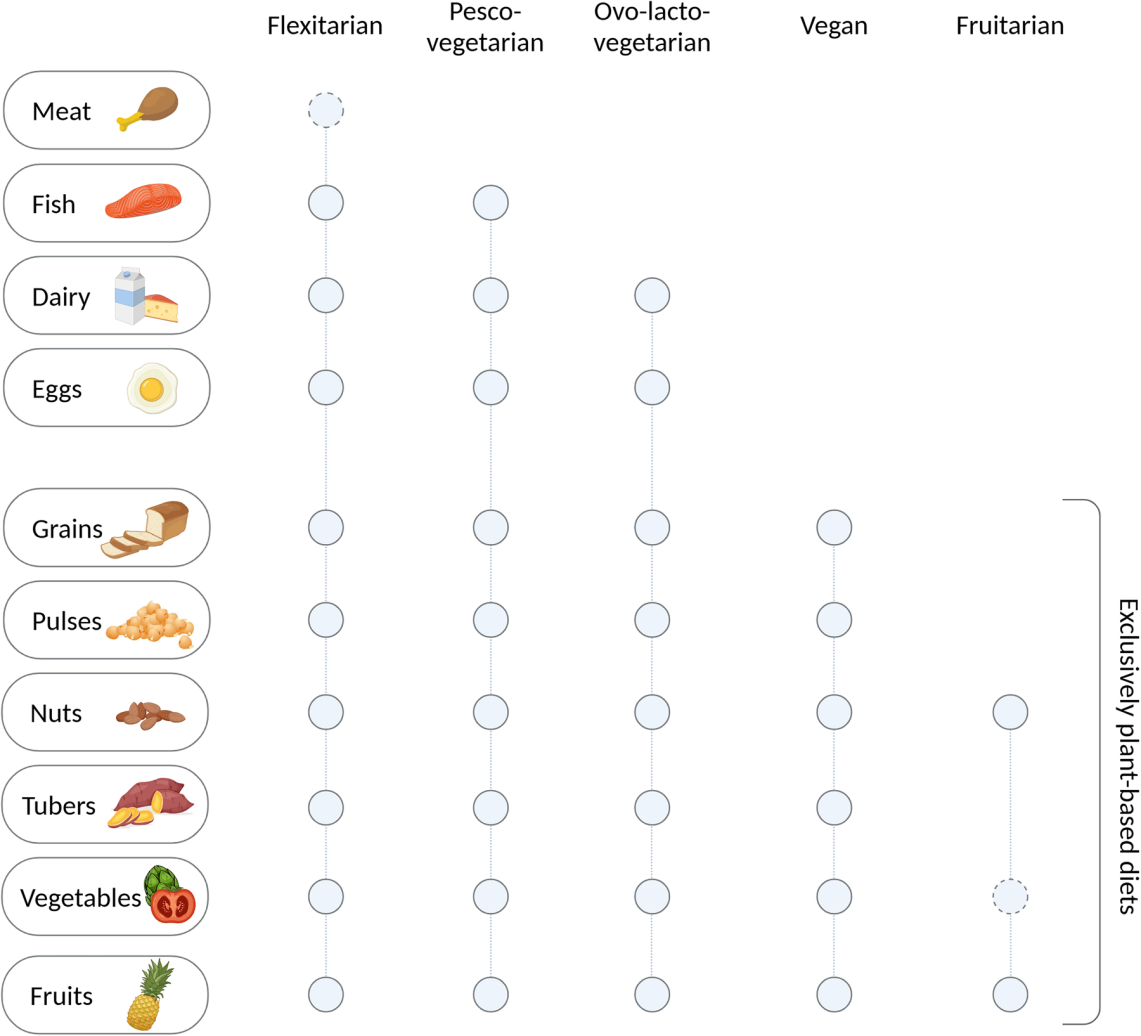
Definitions of plant‐based diets. Flexitarian: plant‐based diet that allows meat consumption in reduced amounts.

There are various types of PBDs and exclusion diets (Figure 1) that may have different impacts on health. Vegetarians exclude meat, meat‐derived foods, and, to different extents, other animal‐derived products.^9^ Some vegetarian diets are classified according to the animal‐derived food source still consumed. For example, pesco‐vegetarians, consume fish and seafood, along with eggs and milk products, and ovolactovegetarians, consume eggs and milk, but avoid meat and fish/seafood^9^ (Figure 1). Vegans follow an even stricter diet in that they exclude all animal‐derived food, for example, honey, animal milk products and eggs.^9^ There are subgroups of veganism that are even more restrictive: for example, a raw food diet is based on unprocessed or uncooked plant‐based foods and avoids food heated >50°C, and a fruitarian diet is purely fruit‐based. Breastfeeding is considered an essential part of early nutrition in vegan infants because, although it is not plant‐based, it does not harm animals. However, plant‐based beverages are gaining popularity amongst vegan parents who are seeking alternative options to traditional cow's milk and dairy‐based infant formulas or drinks. According to a survey by Euromonitor International, 3.4% of Europeans reported following a vegan diet in 2021.^10^ The percentage may vary from 1.8% to 4.5% of the population in Western Europe, the United States, and Australia; however, it is not known how strictly the paediatric age group adheres to a vegan diet.^11^

A vegan diet is not complete and requires micronutrient supplements to avoid deficiencies.^11^ Medical/dietetic societal position papers from North America have stated that well‐planned and adequately supplemented vegan diets are appropriate for all age groups, but this statement was based on little available evidence, and many paediatricians in Europe deem a purely vegan diet unsuitable for children.^12^ Several factors may contribute to different perceptions of how appropriate vegan dietary patterns are, such as socio‐cultural factors and the type of exclusionary diet. All scientific societies agree that vitamin B12 is an essential nutrient that is deficient in a truly vegan diet since it is obtained from animal foods, for example, fish, dairy products and eggs. Vegans can obtain vitamin B12 from fortified processed foods, for example, breakfast cereals and certain nutritional supplements.^13^ According to a recent guideline from the UK National Institute for Health and Care Excellence (NICE), the diagnosis of suspected vitamin B12 deficiency can be made by laboratory measurement of total B12, serum cobalamin or serum holotranscobalamin (holoTC), the active form of B12. A serum cobalamin level of <180 ng/L or holoTC <25 pmol/L confirms B12 deficiency. The result should be considered indeterminate and suggestive of possible vitamin B12 deficiency if serum levels are 180–350 ng/L for cobalamin or 25–70 pmol/L for holoTC.^14^ NICE guidance also advises considering measuring plasma homocysteine or serum methylmalonic acid (MMA) in people who have symptoms or signs of vitamin B12 deficiency and an indeterminate total or active B12 test result.^14^

Another concern about vegan diets is that both the quantity and quality of dietary protein may be lower than in omnivorous diets.^15^ Both the digestibility and essential amino acid content of plant proteins are lower than that of animal proteins.^13^ However, purified or concentrated vegetable proteins, for example soy protein, and gluten have high digestibility (>95%), which is similar to animal proteins. Some intact vegetable products, such as whole cereals and pulses, have a slightly lower protein digestibility of approximately 80%–90%, whereas most other vegetable proteins have an even lower digestibility of 50%–80%.^13^ In view of the lower availability of plant protein, the Italian position paper suggests that vegan children and adolescents may need to consume more protein than recommended for omnivores/the general population.^13^

Paediatric Society Statements are summarised in Table 1. The statements were all expert recommendations based on narrative reviews of the evidence.^13^, ^16^, ^17^, ^18^, ^19^ In 2017, a European Society for Paediatric Gastroenterology, Hepatology and Nutrition (ESPGHAN) position paper on complementary feeding recommended that all infants old enough to start complementary feeding should receive iron‐rich foods, including meat.^20^ The authors also recommended that vegan diets should not be used in infancy/when starting complementary feeding without appropriate medical and dietetic supervision. The literature evaluating whether vegan diets are appropriate for children is limited, not recent and lacking in evidence.^21^ This study focuses specifically on vegan diets in paediatric age, as this dietary pattern is the most restrictive within the plant‐based spectrum, is increasing in popularity, and presents unique nutritional challenges that warrant specific attention. The aim of this ESPGHAN Nutrition Committee position paper was to systematically review any new evidence for the effect of a vegan diet on body growth, nutritional adequacy and laboratory biomarkers in infants, children and adolescents, when compared to children on an omnivorous diet. The practicalities of how to properly plan vegan diets for children will be covered in a second part, as a separate manuscript.

**Table 1:** Previous position statements of relevant nutrition societies regarding vegan and vegetarian diets.

Country	Article – Society	Year	Statement on vegetarian and vegan diets in children
Canada	Vegetarian diets in children and adolescents. Position statement of the Canadian Paediatric Society^16^	2009	Well‐planned vegetarian and vegan diets with appropriate attention to specific nutrient components can provide a healthy alternative lifestyle at all stages of intrauterine, infant, child and adolescent growth.
United States	Position of the Academy of Nutrition and Dietetics: Vegetarian diets^17^	2016	Vegetarian diets provide adequate nutrient intakes for all stages of life. Consuming balanced vegetarian diets early in life can establish healthful lifelong habits. Protein needs of vegan children may be slightly higher than those of non‐vegan children because of differences in protein digestibility and amino acid composition.
Italy	Position paper on vegetarian diets from the working group of the Italian Society of Human Nutrition^13^	2017	Well‐planned vegetarian diets that include a wide variety of plant‐based foods and a reliable source of vitamin B12 provide adequate nutrient intake.* * Although discussing paediatric age in the main text, the final statement does not specifically address children.
Germany	Vegetarian diets in childhood and adolescence. Position paper of the nutrition committee, German Society for Pediatrics and Adolescent Medicine (DGKJ)^18^	2019	A balanced, omnivorous diet with ample consumption of plant‐based foods and moderate consumption of meat, fish and milk products is the recommended diet for children because nutrient requirements are most easily and most likely met. Restrictive diets are associated with an increased risk of nutritional deficiency: the stricter the diet, the greater the risk.
Spain	Position paper on vegetarian diets in infants and children. Committee on Nutrition and Breastfeeding of the Spanish Pediatrics Association^19^	2019	Although following a vegetarian diet is not necessarily unsafe at any point during childhood or adolescence, it is preferable to recommend an omnivorous diet or, at least, an ovo‐lacto‐vegetarian diet during infancy and early childhood.

*Note*: For all the reported position statements, the expert recommendations were based on a narrative review of the evidence.

## 2 METHODS

We conducted a literature review using a systematic search in accordance with the Preferred Reporting Items for Systematic Reviews and Meta‐Analysis statement.^22^

### 2.1 Data sources and search strategies

The literature search was conducted on three databases, including MEDLINE/PubMed, EMBASE and Cochrane Library. The complete search strategy terms are listed in Table S1.

### 2.2 Inclusion and exclusion criteria

The review was developed and structured according to the PICO (Population, Intervention, Control, Outcome) methodology. As summarised in Table 2, the PICO questions addressed were:

1. Are there differences in body growth and anthropometric parameters in children on a vegan diet compared to those on an omnivorous diet?
2. Are vegan children at greater risk of nutritional inadequacies compared to omnivorous children?
3. Are there differences in laboratory biomarkers in vegan children compared to omnivorous children?

**Table 2:** PICO questions.

Topic	Narrative question	P	I	C	O
Vegan diet Outcome A	*Are there differences in body growth and anthropometric parameters in children on a vegan diet compared to those on an omnivorous diet?*	Infants, children and adolescents aged 0–18 years	Vegan diet	Omnivorous diet	Body growth and collection of anthropometric parameters defined as: Weight‐for‐age SDS Length/height‐for‐age SDS Weight‐for‐length SDS BMI‐for‐age SDS Mid upper arm circumference and related SDS Fat mass and fat‐free mass percentages and indexes Tricipital and supra‐iliac skinfold SDS Wasting (weight‐for‐length or BMI‐for‐age <−2 SDS according to WHO growth standards, or other population growth reference) Stunting (height‐for‐age <−2 SDS according to WHO growth standards, or other population growth reference) Low body weight (weight‐for‐age <−2 SDS according to WHO growth standards, or other population growth reference) Overweight (BMI‐for‐age >+2 SDS between 2 and 5 years of age or BMI‐for‐age >+1 SDS after 5 years of age, according to WHO growth charts, or other population growth reference) Obesity (BMI‐for‐age >+3 SDS between 2 and 5 years of age or BMI‐for‐age >+2 SDS after 5 years of age, according to WHO growth charts, or other population growth reference)
Vegan diet Outcome B	*Are vegan children at greater risks of nutritional inadequacies compared to omnivorous children?*	Infants, children and adolescents aged 0–18 years	Vegan diet	Omnivorous diet	Dietary intakes and nutritional adequacy were compared to the EFSA DRVs, or the EAR of the IOM of the US National Academies, for the following items: Energy Macronutrients defined as g/day or % of total energy intake (carbohydrates, sugars, fibres, proteins, fats, saturated fats, polyunsaturated fats, monounsaturated fats, linoleic acid, alpha‐linolenic acid, EPA, DHA) Micronutrients (group B vitamins, vitamin A, vitamin D, vitamin E, vitamin C, vitamin K, calcium, iron, magnesium, zinc, phosphorus, iodine, selenium)
Vegan diet Outcome C	*Are there differences in laboratory biomarkers in vegan children compared to omnivorous children?*	Infants, children and adolescents aged 0–18 years	Vegan diet	Omnivorous diet	Laboratory biomarkers: Blood lipid profile and cardio‐metabolic health Thyroid function Calcium status and bone‐health markers, iron status, ferritin, RBC count, haemoglobin, plasma homocysteine, plasma vitamin B12, whole blood vitamin B12, holoTC, plasma and urinary MMA

Abbreviations: BMI, body mass index; DHA, docosahexaenoic acid; DRV, dietary reference value; EAR, estimated average requirement; EFSA, European Food Safety Authority; EPA, eicosapentaenoic acid; holoTC, holotranscobalamin; IOM, Institute of Medicine; MMA, methylmalonic acid; PICO, population, intervention, comparison/control and outcome; RBC, red blood cell; SDS, standard deviation score; WHO, World Health Organization.

Our systematic search focused on studies investigating vegan diets. Original studies that examined the impact of vegan diets in infants, children, and/or adolescents, published from January 2008 to October 2023 and written in English, were included. The study design was limited to observational studies, cohort studies and trials. We included studies that reported on children following vegetarian diets in addition to those on a vegan diet to compare any differences between the two groups (data from different subgroups were recorded separately). In this review, dietary patterns were classified as follows: a vegan diet excludes all animal‐derived products; a vegetarian diet excludes meat and meat‐derived products, with or without the inclusion of dairy and/or eggs; and an omnivorous diet includes both plant‐based and animal‐based foods. Available systematic reviews and meta‐analyses were also retrieved to expand the discussion. We excluded studies on children with mixed dietary patterns, where vegetarianism and veganism were included as one group. We excluded case reports, case series and narrative reviews and studies in populations with specific diseases.

### 2.3 Identification of relevant studies

**Figure 2:**
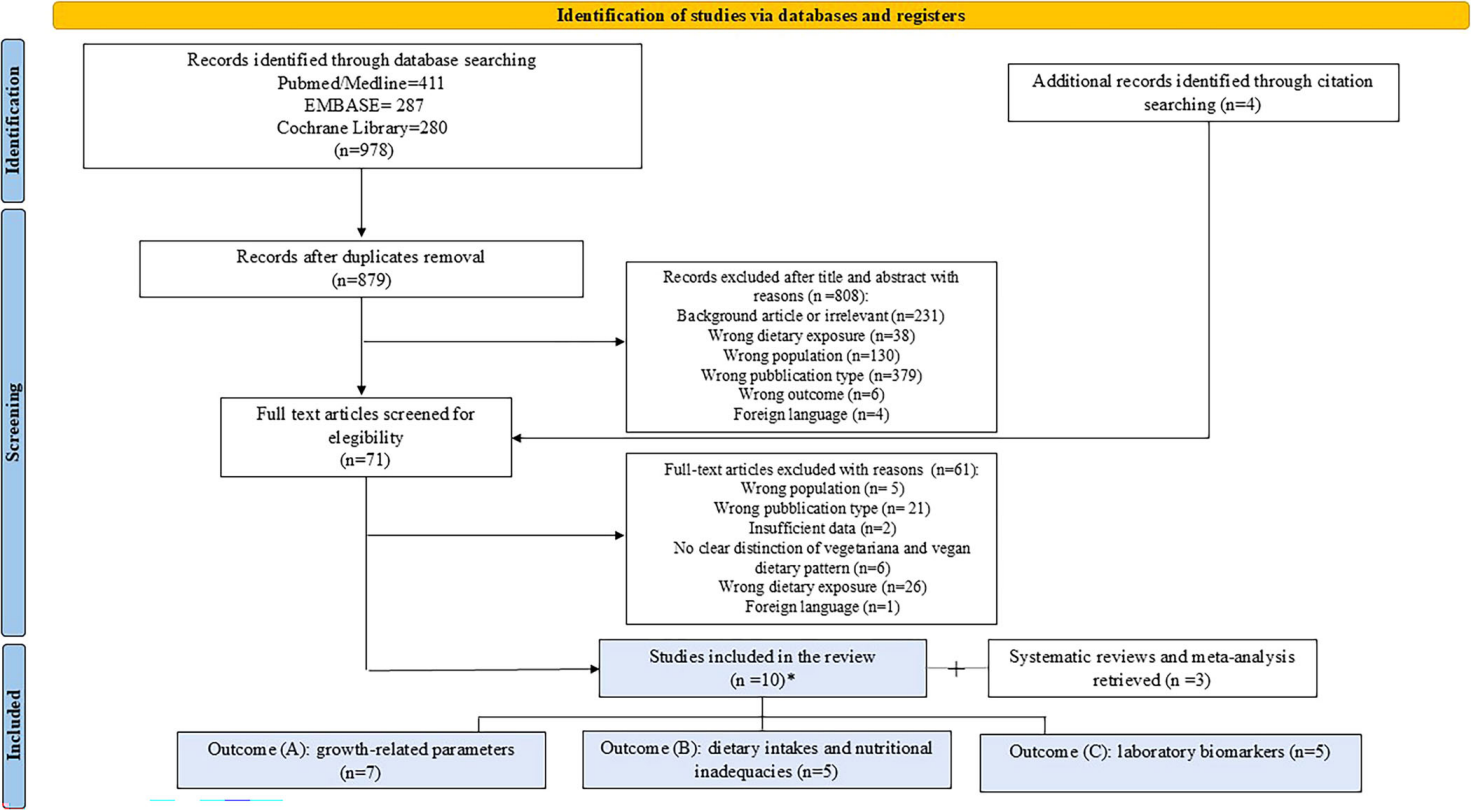
Flowchart of the systematic search. *Some articles belong to more than one outcome.

Study selection was performed by two authors independently (double‐blinded). Rayyan web‐based software was used for reference management, de‐duplication and subsequent screening of the articles.^23^ After the removal of duplicate records, all articles were screened according to their titles and abstracts. Articles were discarded if they failed to meet inclusion criteria (background articles or irrelevant, wrong dietary exposure, wrong population, wrong publication type, wrong outcome and foreign language). Afterwards, full‐text selection was performed, and in case of disagreement about the eligibility of any articles, the opinion of a third author was obtained. Figure 2 reports reasons for full‐text exclusion, which included papers with no clear distinction between vegetarian and vegan diets. In addition, reference lists of the selected studies were searched manually to identify further potentially eligible publications.

### 2.4 Data extraction and interpretation

Data extraction was performed independently and in parallel by two authors. Selected articles were fully analysed to extract the following information: authors, geographical location, publication date, study design, age of participants, reported dietary patterns, sample size and study arms, assessment of dietary intakes, provision of supplements, outcomes assessed and method of assessment, results and confounding factors (if any). Patients were categorised into age groups as follows: infants (<12 months), toddlers (12–36 months), children (up to 10 years) and adolescents (>10 years). For all the included articles, the exposure was defined as the adoption of a vegan diet. Three outcomes were identified:

– Outcome (A): related to growth we extracted data on specific growth parameters, including, height, weight, body mass index, weight‐for‐length and related standard deviation score (SDS), skinfold thicknesses, mid‐upper arm circumference (MUAC), fat mass and fat free mass percentages and index, as well as risk of stunting, wasting, low body weight for age, and overweight or obesity.
– Outcome (B): information collected on dietary intake included energy and the following nutrients: protein, carbohydrates, sugars, fibre, fats, saturated fatty acids (SFAs), monounsaturated fatty acids (MUFAs), polyunsaturated fatty acids (PUFAs), linoleic acid alpha‐linolenic acid (ALA), eicosapentaenoic acid (EPA), docosahexaenoic acid (DHA), vitamin A, C, D, E, K, group B vitamins, iron, zinc, calcium, iodine, magnesium, selenium and phosphorus. We recorded the adequacy of dietary intake by looking at European Food Safety Authority (EFSA) or the Estimated Average Requirement (EAR) of the Institute of Medicine (IOM) of the US National Academies dietary reference values.^24^, ^25^
– Outcome C: we looked at objective measures of biomarkers related to nutritional status. Data on nutritional biomarkers and health‐related parameters, including calcium status, bone‐health markers, thyroid function, iron status, including ferritin, haemoglobin level for anaemia, blood lipid profile for cardio‐metabolic health, vitamin B12 levels and any other micronutrients assessed, were extracted. We used the World Health Organization (WHO) criteria for iron status and anaemia to interpret results in different studies.^26^, ^27^ Blood lipid profile was assessed according to the reference values reported in the Expert Panel on Integrated Guidelines for Cardiovascular Health and Risk Reduction in Children and Adolescents.^28^

Synthesis of the included articles was collated in tables offering a clear distinction between the vegan group and other diets identified for comparison.

## 3 RESULTS

The initial systematic search retrieved 978 articles (Figure 2). Duplicate records were excluded, and the titles and abstracts of the remaining 808 were screened. We excluded articles that were irrelevant/children were not on a vegan diet, exclusively vegetarian, macrobiotic or fruitarian, certain types of publication, for example, case reports, case series, and narrative reviews, non‐paediatric age‐groups, such as adults, elderly, pregnant women, non‐English language articles, and if the outcomes were not relevant for our search (see selected outcomes in Table 2). There were 71 remaining articles evaluated by reviewing the full text. A total of 10 articles were accepted and included in the final review.^29^, ^30^, ^31^, ^32^, ^33^, ^34^, ^35^, ^36^, ^37^, ^38^ Seven articles were included specifically to assess growth in outcome A, five for nutritional adequacy in outcome B, and five for laboratory investigations in outcome C. The flowchart of the literature search and exclusion process is shown in Figure 2, and the qualitative synthesis of the articles selected is presented in Tables 3, 4, 5, according to the outcome under investigation. The studies gave information on approximately 1500 children who followed a vegan diet. In addition, three systematic reviews and meta‐analyses were retrieved from the systematic search and included.^15^, ^39^, ^40^

**Table 3:** Studies included in the qualitative synthesis for outcome (A): Growth‐related parameters.

Author, year, country	Study design	Population description	Dietary pattern of comparison	Size of groups and study arms	Additional information	Dietary assessment method short	Outcome measures	Methods	Results	Confounding factors assessed
Alexy et al., 2021, Germany^29^	Cross‐sectional study	Children & Adolescents aged 5.5–19 years (mean age 12.7 ± 3.9)	OM and VEG	149 VEG, 115 VN and 137 OM	German VeChi Youth Study	3‐day weight diary	Anthropometry	Anthropometry collection at visit, height and weight not standardised by age and sex	VN vs. VEG and OM (all comparisons): No significant differences between groups for height (cm), weight (kg), and BMI SDS.	Analysis not adjusted for confounding factors; weight and height not presented nor compared as *z*‐scores.
Desmond et al., 2021, Poland^30^	Cross‐sectional study	Children aged 5–10 years	OM	63 VEG, 52 VN, 72 matched OM	Before recruitment, parents completed a screening questionnaire to classify children into groups: VN, VEG and OM. Study did not accept pesco‐vegetarians and semi‐vegetarians.	4‐day weight diary	Anthropometry, body composition	Anthropometry, deuterium dilution, DXA	VN vs. OM: < Height SDS, < BMI SDS, < FMI SDS, < triceps and suprailiac skinfold SDS, < hip and thigh girth SDS. No differences in lean mass index SDS.	Diet group, maternal height, paternal height, birthweight (fifths), gestational age (fifths), maternal pre‐pregnancy BMI (fifths), average movement count per hour internal *z*‐score, breastfeeding duration (<6, 6–12, >12 months), maternal education, paternal education, area of residence (multiple models).
Hovinen et al., 2021, Finland^31^	Cross‐sectional study	Toddlers & Children aged 13–50 months (mean age 3.5 years)	OM and VEG	10 VEG, 6 VN, 24 OM	Vegan participants with a vegan diet since birth (breastfed for 13–50 months by vegan mothers) and weaned more than a year before the study.	4‐day weight record	Anthropometry	Anthropometric measurements.	VN vs. OM; VN vs. VEG (all comparisons): No differences between groups in height, BMI, or MUAC *z*‐scores.	Age and gender.
Pandey, 2021, India^32^	Cross‐sectional study	Infants & Toddlers aged 6–23 months (mean age 16 months)	VEG (Minimal adequacy group is the vegan‐like pattern)	Sample of 5772 (1256 maximum diet adequacy VEG‐like pattern, 4064 medium adequacy VEG‐like pattern, 452 minimum adequacy VN‐like pattern)	National Family Health Survey 2015–2016 (NFHS‐4),	24‐h recall of food	Anthropometry	Stunting, wasting and underweight assessment through WHO growth standards	VN vs. VEG VN had 1.37 times the risk of stunting (95% CI, 1.09–1.7), 1.37 times the risk of wasting (95% CI, 1.05–1.78) and 1.7 times the risk of being underweight (95% CI, 1.35–2.15) in crude models. After adjustments only risk of underweight remained significant. Associations with stunting were more pronounced for non‐breastfed (OR, 2.44; 95% CI, 1.19–5.00) compared to breastfed infants (OR, 1.35; 95% CI, 1.04‐1.74; *p* value for the interaction = 0.07). Associations with wasting were more pronounced among non‐breastfed (OR, 2.82; 95% CI, 1.34–5.95) than breastfed (OR, 1.12; 95% CI, 0.85–1.5; *p* value for the interaction = 0.03).	Socioeconomic status (maternal education level), birth order, sex, and age group.
Svetnicka et al., 2022, the Czech Republic^33^	Cross‐sectional study	Infants, Toddlers, Children & Adolescents Aged 0.5–18.5 years (4.8 ± 5.8 mean years for VN)	OM and VEG	79 VEG, 69 VN and 52 OM (subgroup infants 13 VEG and 34 VN)	Czech Vegan Children Study (CAROTS). Subgroup analysis (*n* = 46) of breastfed children (0–3 years) from VEG or VN mothers.	3‐day weight record	Anthropometry	Anthropometry with the Czech Republic standard percentiles	VN vs. VEG and OM (all comparisons): No differences in height percentile, weight percentile, or BMI percentile. VN vs. OM Higher number of VN children with BMI‐for‐age <3 percentile (*p* = 0.003).	Age and sex.
Weder et al., 2019, Germany^34^	Cross‐sectional study	Toddlers aged 1–3 years	OM and VEG	139 VN, 127 VEG and 164 OM children	German VeChi Youth Study and DONALD (DOrtmund Nutritional and Anthropometric Longitudinally Designed)	3‐day weight record	Anthropometry	An online questionnaire assessed BW and height. Stunting, wasting, and underweight assessment through WHO growth standards	VN vs. OM; VN vs. VEG (all comparisons): No differences in weight‐for‐height, height‐for‐age, weight‐for‐age SDS between the groups. Percentage of stunted children VN 3.6%, compared with VEG 2.4% and OM 0%. Percentage of children with overweight or at risk of overweight: VN 18% vs. VEG 18.1% vs. OM 23.2%.	Age, sex, breastmilk intake, socioeconomic status, physical activity, weight‐for‐height *z*‐score, paternal BMI, seasons, and urbanicity (multiple models).
Wirnitzer, 2021, Austria^35^	Cross‐sectional study	Adolescents aged 15.1 ± 2.3 years	OM and VEG	VN 633, VEG 745, OM 7421	Science 2 School Austrian nationwide survey	Self‐reported questionnaire	Patient‐reported anthropometric parameters and BMI classification	German reference values with the 90th and 97th percentiles serving as cut‐off points for overweight and obesity, or below the 10th percentile as the underweight cut‐off	VN vs. OM: < height (cm), < BW (kg). No differences for BMI percentiles. Prevalence of underweight 15% VN vs. 11.9% OM (*p* < 0.01). Prevalence of normal weight 72% VN vs. 76.1% OM (*p* < 0.01). No difference in obesity prevalence (4.9% both groups). VN vs. VEG: < height (cm), < weight (kg), > BMI percentile. Prevalence of overweight no different. Prevalence of obesity 4.9% VN vs. 2.6% VEG (*p* < 0.01).	Environment (urban vs. rural), sex, school type and sport.

Abbreviations: BMI, body mass index; BW, body weight; CI, confidence interval; FMI, fat mass index; MUAC, mid‐upper arm circumference; OM, omnivorous; OR, odds ratio; SDS, standard deviation score; VEG, vegetarian; VN, vegan; WHO, World Health Organization.

**Table 4:** Studies included in the qualitative synthesis for outcome (B): Dietary intakes and nutritional inadequacies.

Author, year, country	Study design	Population description	Dietary pattern of comparison	Size of groups and study arms	Groups additional information	Dietary assessment method	Supplements	Outcome measures	Methods	Results	Confounding factors assessed
Alexy et al., 2021, Germany^29^	Cross‐sectional study	Children & Adolescents aged 5.5–19 years (mean age 12.7 ± 3.9)	OM and VEG	149 VEG, 115 VN and 137 OM	German VeChi Youth Study	3‐day weight diary	Yes, *n* = 201 (52.4% participants supplemented – e.g., Vit B12 – Vit D – iron – folate)	Dietary intakes	Dietary supplements not considered. Nutrient intake calculation focuses on nutrient intake from food.	VN vs. VEG: No diff energy intake; < energy density (*p* = 0.0039). < sugars%, > fibre, < SFA%, > PUFA% (*p* = 0.0002); > protein g/kg/day (*p* = 0.018); < FAT% (*p* = 0.037). Median B12 intake (μg/1000 kcal) was 0.1 (0.0; 0.2 IQR) > Vit E; > Vit C; < folate; >B1; <B2 (*p* < 0.01). < calcium; > magnesium; > iron; > zinc (mg/1000 kcal) (*p* < 0.01). VN vs. OM: No diff. energy intake or energy density > CHO%, < sugars%, > Fibre, < FAT%, < MUFA%, < SFA%. > PUFA% (*p* < 0.01); no differences for proteins > Vit E; > Vit C; > folate; >B1; <B2 (*p* < 0.01). < calcium, > magnesium, > iron (mg/1000 kcal). German reference values: Median protein intake VN 1.16 g/BW/day (0.89–1.67 IQR) > recommendations 0.9 g/BW/day. Vit B12 intake: 4% VN, 32% VEG, 86% OM of German reference values.	Age (years), BMI‐SDS, socioeconomic status (low/middle/high), smoking in the household (yes/no), physical activity (MET‐minutes), use of dietary supplements (yes/no).
Hovinen et al., 2021, Finland^31^	Cross‐sectional study	Toddlers & Children aged 13–50 months (mean age 3.5 years)	OM and VEG	40 children (10 VEG, 6 VN, 24 OM),	Children on a vegan diet since birth (breastfed for 13–50 months by vegan mothers) and weaned >1 year before the study.	4‐day weight record	YES (5/6VN subject Vit B12 and iodine supplemented, 6/6VN Vit D supplemented)	dietary intakes		VN mean protein intake %En: 13.5 [10.1–16.4 range]. VN mean iron intake (mg/day) 11.9 [10.6–15.1 range]. VN vs. OM: No different energy intake < Protein %En, < SFA%, < cholesterol (g/day), < EPA and DHA. Vitamin B12 all supplemented apart from 1 [with supplements 43 μg/day vs. without supplementation VN 3.2 μg/day vs. OM 3.5 μg/day] > MUFA%, > PUFA%, > LA and > ALA, > fibre, > Vit B1 and > Vit B2 and > Vit B3, > folate (μg/day), > Vit E, > Vit K, > phosphorus, > zinc (mg/day), > iron (mg/day) (also without supplements) Vit B12 VN with supplements 3 μg/day vs. 3.8 μg/day in OM. VN vs. VEG: No different energy intake < cholesterol (g/day), < DHA, < folate (μg/day), > Vit B2.	Age and gender.
Weder et al., 2019, Germany^34^	Cross‐sectional study	Toddlers aged 1‐3 years	OM and VEG	139 VN, 127 VEG and 164 OM	German VeChi Youth Study and DONALD (DOrtmund Nutritional and Anthropometric Longitudinally Designed)	3‐day weight record	NA	dietary intakes		No significant differences for energy intake or density among groups. The median protein intake in VN was 2.25 g/BW/day (1.82–2.76 IQR). VN vs. OM vs. VEG: < median intakes of protein g/kg (OM: 2.7, VEG: 2.3, VN: 2.4 g/kg BW, *p* < 0.0001), < fat %En (OM: 36.0, VEG: 33.5, VN: 31.2, *p* < 0.0001), and < added sugars %En (OM: 5.3, VEG: 4.5, VN: 3.8, *p* = 0.002), > CHO %En (OM: 50.1, VEG: 54.1, VN: 56.2%, *p* < 0.0001) and > fibre g/1000 kcal (OM: 12.2, VEG: 16.5, VN: 21.8, *p* < 0.0001) [*p* values among groups].	Age, sex, breastmilk intake, SES, physical activity, weight‐for‐height *z*‐score, paternal BMI, seasons and urbanicity (multiple models).
Weder et al., 2022 (a), Germany^36^	Cross‐sectional study	Toddlers & Children aged 0.9–4.3 years	OM and VEG	139 VN, 127 VEG and 164 OM	German VeChi Youth Study and DONALD (DOrtmund Nutritional and Anthropometric Longitudinally Designed)	3‐day weight record	YES (all supplements 135/139 VN, Vit B12 supplement 135/139)	Dietary intakes	Comparison of dietary intake among different groups with or without supplementation.	VN Median iron intake (mg/day) was 8.9 (6.0–11.6 IQR) without supp and 9.2 (6.1–11.9 IQR) with supplements. Median Vit B12 intake (μg/day) was 0.2 (0.1–0.4 IQR) without supp and 71.4 (5.5–333.5 IQR) with supplementation. VN vs. OM: Higher fat quality intake (>PUFA%, > LA and > ALA and < SFA%). OM children had the highest intake of SFA, AA, EPA, DHA, and cholesterol. > intake of vitamin E, K, B1, B6, C, folate, magnesium, potassium and iron (without supplements). < intake of vitamin B2, B12, calcium, iodine (without supplements). With supplements, same results and also < EPA and < DHA, whilst > Vit B12 and > Vit D. Vit B12 in VN with supplements 73.8 μg/day versus 1.7 μg/day in OM. VN vs. VEG: > Vit E, > Vit K, > Vit B1, > vit B6, > folate, > potassium, > magnesium, > iron, < iodine, < Vit B12. < SFA, > PUFA%, > LA, > ALA, > EPA, > DHA, < cholesterol. With supplements, same results apart from no difference for iodine, > Vit B12 and > Vit D in VN. All groups vs. h‐AR. Without supplementation, none of the groups' median intakes met the h‐AR for Vit D and iodine. VEG and VN children did not achieve h‐ARs for Vit B2, Vit B12, and iron; VN children also did not do so for calcium.	Age, sex, breastmilk intake, socioeconomic status, physical activity, energy intake, weight‐for‐height *z*‐score, paternal BMI, seasons, and urbanicity (multiple models).
Weder et al., 2022 (b), Germany^37^	Cross‐sectional study	Toddlers aged 1–3 years	OM and VEG	139 VN, 127 VEG and 164 OM	German VeChi Youth Study and DONALD (DOrtmund Nutritional and Anthropometric Longitudinally Designed)	3‐day weight record	YES (2.4% of VEG, 11.5% of VN, and 0.6% of OM took a selenium or selenium‐containing supplement)	dietary intakes	The selenium intake was assessed and Assessment of h‐AR to estimate the percentage of subjects at risk of nutrient deficiency.	The median daily selenium intake was 17 μg, 19 μg, and 22 μg in VEG, VN and OM children, respectively. VN vs. OM: < selenium μg/day and < selenium μg/1000 kcal/day. 52% VEG, 47% VN, 28% OM had selenium intake below h‐AR (17 μg/day), while 39% VEG, 36% VN, 16% OM had a selenium intake of <15 μg/day (adequate intake EFSA).	Adjusting for age, sex, weight‐to‐height *z*‐scores, season, socioeconomic status, and total energy intake.

Abbreviations: ALA, alpha‐linolenic acid; BMI, body mass index; BW, body weight; CHO, carbohydrates; DHA, docosahexaenoic acid; EFSA, European Food Safety Authority; EPA, eicosapentaenoic acid; h‐AR, harmonised average requirement; IQR, interquartile range; LA, linoleic acid; MUFA, monounsaturated fatty acid; OM, omnivorous; PUFA, polyunsaturated fatty acid; SDS, standard deviation score; SFA, saturated fatty acid; Vit, vitamin; VEG, vegetarian; VN, vegan.

**Table 5:** Studies included in the qualitative synthesis for outcome (C): Nutritional biomarkers and health‐related parameters.

Author, year, country	Study design	Population description	Dietary pattern of comparison	Size of groups and study arms	Groups additional information	Dietary assessment method short	Supplements info	Outcome measures	Methods	Results	Confounding factors assessed
Alexy et al., 2021, Germany^29^	Cross‐sectional study	Children & Adolescents aged 5.5–19 years (mean age 12.7 ± 3.9)	OM and VEG	149 VEG, 115 VN and 137 OM	German VeChi Youth Study	3‐day weight diary	Yes, *n* = 201 (52.4% participants supplemented)	Nutrient biomarker + blood lipid concentrations	Vitamin B12 deficiency likely: holoTC < 35 pmol/L and MMA > 271 nmol/L Vitamin B2: deficiency FAD < 199 μg/L	VN vs. OM < ferritin (*p* = 0.04), < Total chol, < LDL, < non‐HDL (*p* = 0.001). VN vs. VEG > folate, < Total chol, < LDL, < non‐HDL (*p* < 0.01). All groups vs. references: – considering holoTC and MMA concentrations, 8% of VN, 13% VEG, 4% OM classified as likely deficient. – 25‐OH vitamin D3 concentrations <20 ng/mL (VEG 36%, VN 27%, OM 28%), and <12 ng/mL (VEG 10%, VN 5%, OM 4%). – low concentrations of vitamin B2 (FAD) (VEG 50%, VN 54%, OM 37%).	Age (years), BMI‐SDS, socioeconomic status (low/middle/high), smoking in the household (yes/no), physical activity (MET‐minutes), use of dietary supplements (yes/no).
Desmond et al., 2021, Poland^30^	Cross‐sectional study	Children aged 5–10 years	OM	63 VEG, 52 VN, 72 matched OM	Before recruitment, parents completed a screener questionnaire to classify children into groups: VN, VEG and OM. Study did not accept pesco‐vegetarians and semi‐vegetarians.	4‐d weight diary	YES (29% VN were not Vit B12 supplemented or given B12– Fortified foods; 32.7%V N were Vit D supplemented.	blood lipids profile, micronutrient status [iron, Vit B12, and 25‐hydroxy vitamin D (25(OH)D)]. bone health, CVD risk markers	Probable and possible vitamin B12 deficiency defined as <148 pmol/L and 148–258 pmol/L, respectively. Carotid ultrasound, fasting blood samples, and accelerometry data DXA	VN vs. OM < Total chol, < HDL, < LDL, < hs‐CRP, > HOMA‐IR – Prevalence of high LDL (≥130 mg/dL) and borderline high LDL (110–129 mg/dL) 13% and 17% for OM and 0% and 1% VN (*p* < 0.05). – Prevalence of low HDL ( < 45 mg/dL) and borderline low HDL (40–45 mg/dL) 7% and 12% for OM, 26% and 24% VN (*p* < 0.05). Worse iron status (<RBC, < haemoglobin, < haematocrit %, < Ferritin%). – Prevalence of mild anaemia 0% OM vs. 6% VN (*p* < 0.05). – Prevalence of depleted iron stores (serum ferritin <15 μg/L) 12.8% OM vs 30.2% VN (*p* < 0.05). <L2–L4 bone mineral content% (adj. model Δ: −5.6 95% CI: −10.6, −0.5); < total body less head bone mineral content% (adj. model Δ: −3.7 95% CI: −7.0, −0.4) (*p* < 0.05). VN not supplemented vs. OM < Vit B12 pmol/L, > homocysteine, > MCV (*p* < 0.01). VN supplemented and fortified vs. OM No difference in Vit B12 profile. No difference in Serum Vit D25(OH). VN only fortification vs OM < Vit B12 pmol/L. – Prevalence of probable Vit B12 deficiency 3% OM vs. 13% VN (*p* < 0.05). – Prevalence of possible Vit B12 deficiency 16% OM vs. 40%VN (*p* < 0.05).	Adjustment for metabolism parameters: diet group; age; sex; birthweight quintile; gestational age quintile; maternal pre‐pregnancy BMI quintile; breastfeeding at 6, 6–12 and >12 mo; maternal education; paternal education; religion; urbanicity; height *z‐*score (UK); fat mass *z*‐score (DXA); lean mass *z*‐score (DXA). Adjustment for iron status: diet group, age, sex, maternal education, urbanicity and maternal smoking. Linear regression was used to test the null hypothesis of no difference between vegetarians and omnivores, as well as vegans and omnivore groups. Adjustment for bone health: diet group, age, sex, maternal education, religion, urbanicity, height *z*‐score (UK), weight *z*‐score (UK), bone area.
Hovinen et al., 2021, Finland^31^	cross‐sectional study	Toddlers & Children aged 13–50 months (mean age 3.5 years)	OM and VEG	10 VEG, 6 VN, 24 OM	Children on a vegan diet since birth (breastfed for 13–50 months by vegan mothers) and weaned more than a year before the study.	4‐d weight record		Nutrient biomarker, blood lipid concentrations, metabolomics and lipidomics	Blood and urine samples and assessment of biomarkers of micronutrient status and cholesterol metabolism	VN vs. OM: < vitamin D, Retinol binding protein, Transthyretin, vitamin A, whilst > folate values. – In all vegan participants, Vitamin A was insufficient, and vitamin D was borderline sufficient. < total, LDL, and HDL cholesterol concentrations > cholesterol absorption biomarkers: cholestanol, campesterol, sitosterol, avenasterol). No differences for biomarkers of cholesterol biosynthesis. > higher steady‐state levels of unconjugated primary bile acids, and < taurine to glycine conjugation ratio of bile acids. No differences for serum ferritin, transferrin receptor, zinc or UIC. Serum concentrations of vitamin B12 were adequate in all groups.	Age and gender.
Svetnicka et al., 2022, Czech Republic^33^	Cross‐sectional study	Infants, Toddlers, Children & Adolescents aged 0.5–18.5 years (4.8 ± 5.8 mean age for VN)	OM and VEG	79 VEG, 69 VN and 52 OM (subgroup infants 13 VEG and 34 VN)	Czech Vegan Children Study (CAROTS). Subgroup analysis (*n* = 46) of breastfed children (0–3 years) from VEG or VN mothers.	3‐day weight record	YES (59/69 VN supplemented with Vit B12, 54/79 VEG)	holoTC, cyanocobalamin, folate, homocysteine, MCV and haemoglobin.	Fasting blood samples	All groups No significant differences in holoTC, folate, homocysteine (hcys) or MCV. Significant difference was found in levels of Vit B12 among groups (VEG 572 vs. OM 432.5 vs. VN 18.1; *p* = 0.019), although only 1 VEG and 2 VN children had vitamin B12 deficiency. Non‐supplemented VN vs. VEG: Non‐supplementing VEGs are at the same risk of vitamin B12 deficiency as non‐supplementing vegans. 35 VEG, 28 VN and 9 OM children had vitamin B12 hypervitaminosis, probably due to over‐supplementation. Vit B12, holoTC, and homocysteine levels significantly higher (*p* < 0.05) among supplemented VEG/VN children compared to the non‐supplemented ones.	Age and sex.
Svetnicka et al., 2023, Czech Republic^38^	Cross‐sectional study	Infants, Toddlers, Children & Adolescents aged 0.5–18.5 y (4.4 ± 5.5 mean age for VN)	OM and VEG	VEG 92, VN 78, 52 OM	Czech Vegan Children Study (CAROTS)	3‐day weight record	YES, and related doses of supplements		Fasting blood samples	VN vs. OM vs. VEG: UIC significantly different (OM: 197.28 ± 105.35 vs. VEG: 177.95 ± 155.88 vs. VN: 162.97 ± 164.51 μg/L; *p* < 0.001). – Prevalence of iodine deficiency (UIC < 100 μg/L) was VN 41.9% vs. VEG 34.8% vs. OM 19.6% (*p* = 0.06). Presence of anti‐thyroglobulin antibodies (AhTGc) more common in VEG (18.2%)/VN (35.0%) groups vs. OM (2.1%) (*p* < 0.001). No significant differences in levels of TSH, FT3, Tg or anti‐thyroid peroxidase antibody (ATPOc) among groups. VN vs. OM > FT4 levels (*p* < 0.001).	Age and sex.

Abbreviations: FAD, flavin adenine dinucleotide; FT3, free triiodothyronine; FT4, free thyroxine; HDL, high‐density lipoprotein cholesterol; holoTC, Holotranscobalamin; HOMA‐IR, Homeostatic Model Assessment for Insulin Resistance; hs‐CRP, high‐sensitivity C‐reactive protein; L2–L4, lumbar spine L2–L4 bone mineral content; LDL, low‐density lipoprotein cholesterol; MCV, mean corpuscular volume; MMA, methylmalonic acid; OM, omnivorous; RBC, red blood cell; Tg, thyroglobulin; TSH, thyroid‐stimulating hormone; UIC, iodine in spot urine; VEG, vegetarian; VN, vegan.

### 3.1 Outcome A: Body growth and anthropometric parameters

#### 3.1.1 Length/height

Among the seven selected studies that looked at growth in height,^29^, ^30^, ^31^, ^32^, ^33^, ^34^, ^35^ one article found significantly lower height SDS^30^ in vegan school‐age children (5–10 years) compared to omnivores, and one showed a lower absolute height (cm) in adolescents, but was not standardised for age and sex.^35^ Pandey et al. found a significantly higher risk of stunting (according to WHO growth references) among vegan infants and toddlers (6–23 months), compared to vegetarians in crude models, but the association lost significance after adjustment.^32^ In contrast, four other studies found no differences in linear growth between vegans and omnivores across different ages.^29^, ^31^, ^33^, ^34^ There was a lack of information concerning participants receiving professional dietary counselling on following a vegan diet.

#### 3.1.2 Weight‐for‐length, body mass index (BMI) and body composition

One study found a significantly lower BMI SDS in vegan children compared with omnivores.^30^ Five others found no significant difference in weight‐for‐height and/or BMI‐for‐age ^31^, ^32^, ^33^, ^34^, ^35^ although two of them recorded a higher prevalence of underweight and/or wasting ^33^, ^35^ and one reported a lower prevalence of obesity ^34^ in vegan children compared to omnivores. Only Desmond et al.'s study assessed other adiposity measures. They found lower values for fat mass index SDS, triceps skinfold and supra‐iliac skinfold SDS compared to omnivorous children without any difference in lean mass index SDS.^30^

It was not possible to detect whether professional nutritional advice influenced weight‐related parameters since the studies did not provide this information.

### 3.2 Outcome B: Dietary intake and nutritional inadequacies

#### 3.2.1 Macronutrients and energy intake

##### 3.2.1.1 Energy intake

Two systematic reviews and three studies evaluated the energy intake of vegan children.^15^, ^29^, ^31^, ^34^, ^39^ They did not find any significant difference in energy intake in vegan children compared to omnivores.

##### 3.2.1.2 Macronutrient intake

Three studies comprehensively reported on macronutrient intake, by means of 3‐ or 4‐day food records.^29^, ^31^, ^34^ Two of the three reported a significantly lower intake of protein by vegans compared to omnivores,^31^, ^34^ while one did not find a significant difference.^29^ Two studies showed that the median intake of protein in vegan children was within the EFSA population reference intake (PRI) recommended per kg body weight per day (kg/BW/day).^29^, ^34^ They did not ascertain whether the amount of protein consumed was sufficient to ensure an optimal nitrogen balance. Based on this, the meta‐analysis of Neufingerl et al. concluded that in almost all the studies, the protein intake met the lower level of the acceptable macronutrient distribution range.^39^ However, in their meta‐analysis, Koller et al. pointed out that, besides protein quantity, protein quality may be lower among vegans.^15^

Two studies reported a higher percentage of daily energy intake (%En) from carbohydrates (CHO) and lower %En from sugars, compared to omnivores.^29^, ^34^ Three studies reported on dietary fibre intakes in vegan children,^29^, ^31^, ^34^ compared to omnivores. In two studies on vegan toddlers and young children, the mean daily fibre intake of vegans exceeded the adequate intake (AI) recommended by EFSA (AI 10 g/day and 14 g/day for 1–3 and 4–6 years of age, respectively).^31^, ^34^ In contrast, in the third study, the fibre intake was within the recommended range in vegan school‐aged children and adolescents (AI ranging from 16 to 21 g/day).^29^

Three studies showed a consistently lower intake of SFA (%En), higher intake of the PUFA (%En),^29^, ^31^, ^34^ ALA (%En) and linoleic acid (LA%En)^31^, ^34^ than omnivorous children and inconclusive results for MUFA (%En).^29^, ^31^ The above findings were confirmed in two meta‐analyses.^15^, ^39^

#### 3.2.2 Micronutrient intake

##### 3.2.2.1 Iron

Dietary mineral content was assessed in four articles.^29^, ^31^, ^36^, ^37^ Three of the four studies evaluated iron intake ^29^, ^31^, ^36^ and confirmed a higher mg/day intake in vegan than in omnivorous children. A meta‐analysis by Neufingerl et al. found that iron intake from food generally met the EAR for the child's age and gender.^39^ How a vegan diet should provide adequate iron intake and subsequent adequate iron metabolism will be summarised in a separate manuscript. The bioavailability of iron in a non‐haem form from plant sources is less than that of haem iron, found in animal food sources.^41^

##### 3.2.2.2 Calcium

Two studies reported lower intake of calcium in vegan children compared to omnivores,^29^, ^36^ with half the vegan group reaching the reference value for calcium. A meta‐analysis found a borderline nonsignificant difference in calcium intake,^15^ whereas most studies included in the Neufingerl et al. meta‐analysis found that total calcium intake, whether from dietary sources alone or combined with supplementation, was below the EAR for age and gender for all dietary patterns, including vegan.^39^

##### 3.2.2.3 Magnesium and zinc

The mean intake of magnesium was above the EAR for age and gender in vegan children^39^ and higher than in children on omnivorous diets.^29^, ^39^ Only one study investigated the difference in zinc intake according to type of diet, without finding any significant difference.^29^

##### 3.2.2.4 Other minerals/trace elements

One study reported a higher intake of potassium and lower intake of iodine in children on vegan diets compared to omnivorous.^36^ Another study reported lower daily selenium intake (μg/day and μg/1000 kcal/day) with a higher percentage of children not meeting the reference AI according to EFSA^37^ when compared to omnivorous children.

##### 3.2.2.5 Vitamin B12

Three studies investigated the dietary intake of vitamin B12, or cobalamin, in vegan compared to omnivorous children.^29^, ^31^, ^36^ In the study from Alexy et al., only 4% of vegan children reached the recommended EFSA AI of vitamin B12 (1.5, 2.5, 3.5 and 4 μg/day for children 1–6, 7–10, 11–14 and 15–17 years of age, respectively).^29^ In the study of Weder et al., unsupplemented vegan children showed that median vitamin B12 intake was 0.2 μg/day (0.1–0.4 μg/day interquartile range), which was below the EFSA AI for children 1–3 years.^36^ Accordingly, the meta‐analysis concluded that vegans usually had an intake below the EFSA DRV.^39^

When analysing the subgroup of vegan children on supplements, two studies reported significantly higher intakes of vitamin B12 compared to omnivorous children (73.8 μg/day vs. 1.7 μg/day in omnivores in the study of Weder et al. or a mean of 43 μg/day vs. 3.8 μg/day in the study of Hovinen et al.).^31^, ^36^ These conclusions are also supported by the Neufingerl et al. ^39^ meta‐analysis.

##### 3.2.2.6 Other vitamins

Overall, vegan children showed a greater intake of folate, vitamin B1 and vitamin E compared to omnivorous children with average intakes exceeding the dietary reference value in all the studies that analysed them,^29^, ^31^, ^36^ a finding that was also seen in one meta‐analysis.^39^ Inconsistent results were obtained for vitamin B2.^15^, ^29^, ^31^, ^36^, ^39^ Two studies reported higher intake of vitamin K in a vegan diet,^31^, ^36^ while one reported higher vitamin C intake compared to a omnivorous diet.^29^ The average intake of both vitamin C and K exceeded reference values in all the studies that measured them.

Although the included articles thoroughly investigated the content of a vegan diet, none of the articles assessed whether children and their families had received dietary counselling. One study reported on the use of fortified foods in addition to supplements.

Dietary assessment in the included studies was often limited and provided poor‐quality data. Thus, estimated intakes of some macro and micronutrients were unreliable.

### 3.3 Outcome C: Nutritional biomarkers and health‐related parameters

#### 3.3.1 Lipid profile

Overall, a healthier blood lipid profile was identified in vegan children compared with omnivores. Vegan children exhibited lower total cholesterol and low‐density lipoprotein (LDL) levels.^15^, ^29^, ^30^, ^31^ There were virtually no borderline high (110–129 mg/dL) and no high LDL (≥130 mg/dL) levels in vegans.^30^ In some studies, vegan children had lower high‐density lipoprotein (HDL) levels,^15^, ^30^, ^31^ and in two studies HDL mean values were borderline‐low (40‐45 mg/dl) according to Expert Panel on Integrated Guidelines for Cardiovascular Health and Risk Reduction in Children and Adolescents.^28^ One study demonstrated higher cholesterol absorption biomarkers in vegan children than in omnivores, while there was no difference in cholesterol biosynthesis biomarkers.^31^

#### 3.3.2 Vitamin B12 status

**Figure 3:**
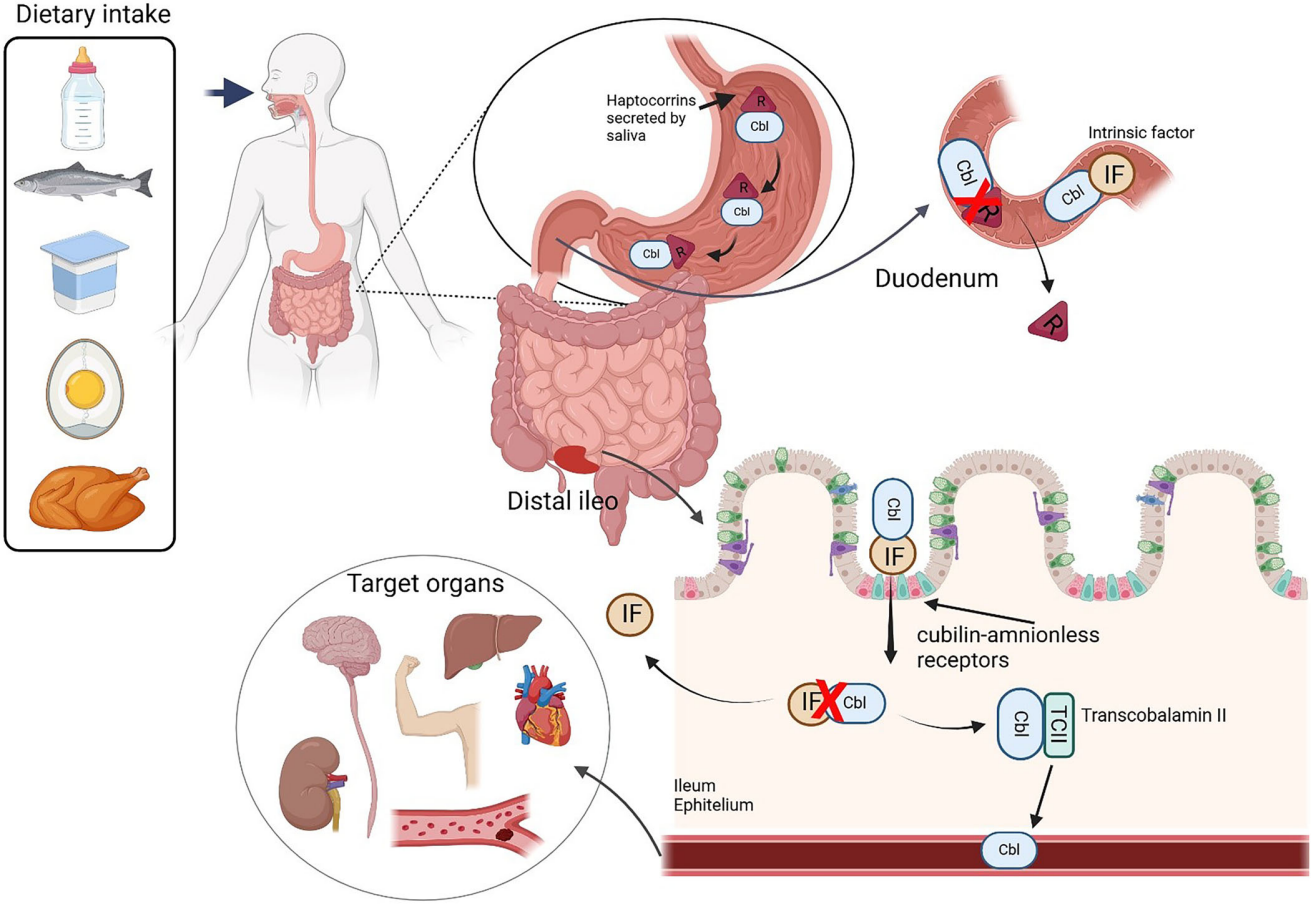
Physiological mechanisms of vitamin B12 absorption. Following the consumption of a meal rich in vitamin B12, including foods such as fish, chicken, milk, yoghurt, and eggs, vitamin B12 or Cbl binds to R secreted by saliva, forming a complex that protects it from degradation. Vitamin B12 in food is initially bound to proteins and is released in the stomach through the action of gastric acid and pepsin. Once freed, the vitamin binds to R‐binders (haptocorrins) secreted by saliva and the gastric mucosa. In the small intestine, pancreatic proteases break down the R‐binders, freeing vitamin B12, which subsequently binds to IF, a glycoprotein produced by parietal cells in the stomach. The resulting vitamin B12‐intrinsic factor complex travels to the distal ileum, where specific receptors recognise and mediate its absorption through receptor‐mediated endocytosis. Within the intestinal epithelial cells, intrinsic factor is degraded, releasing vitamin B12 into the intercellular flow bound to TCII, a transport protein. This complex is then transported via the bloodstream to tissues, particularly the liver, which serves as the primary storage site, holding up to 3 mg of vitamin B12. Efficient enterohepatic recycling further minimises losses, ensuring the body's vitamin B12 supply is maintained. Cbl, cobalamin; IF, intrinsic factor; R, haptocorrins; TCII, transcobalamin II.

**Figure 4:**
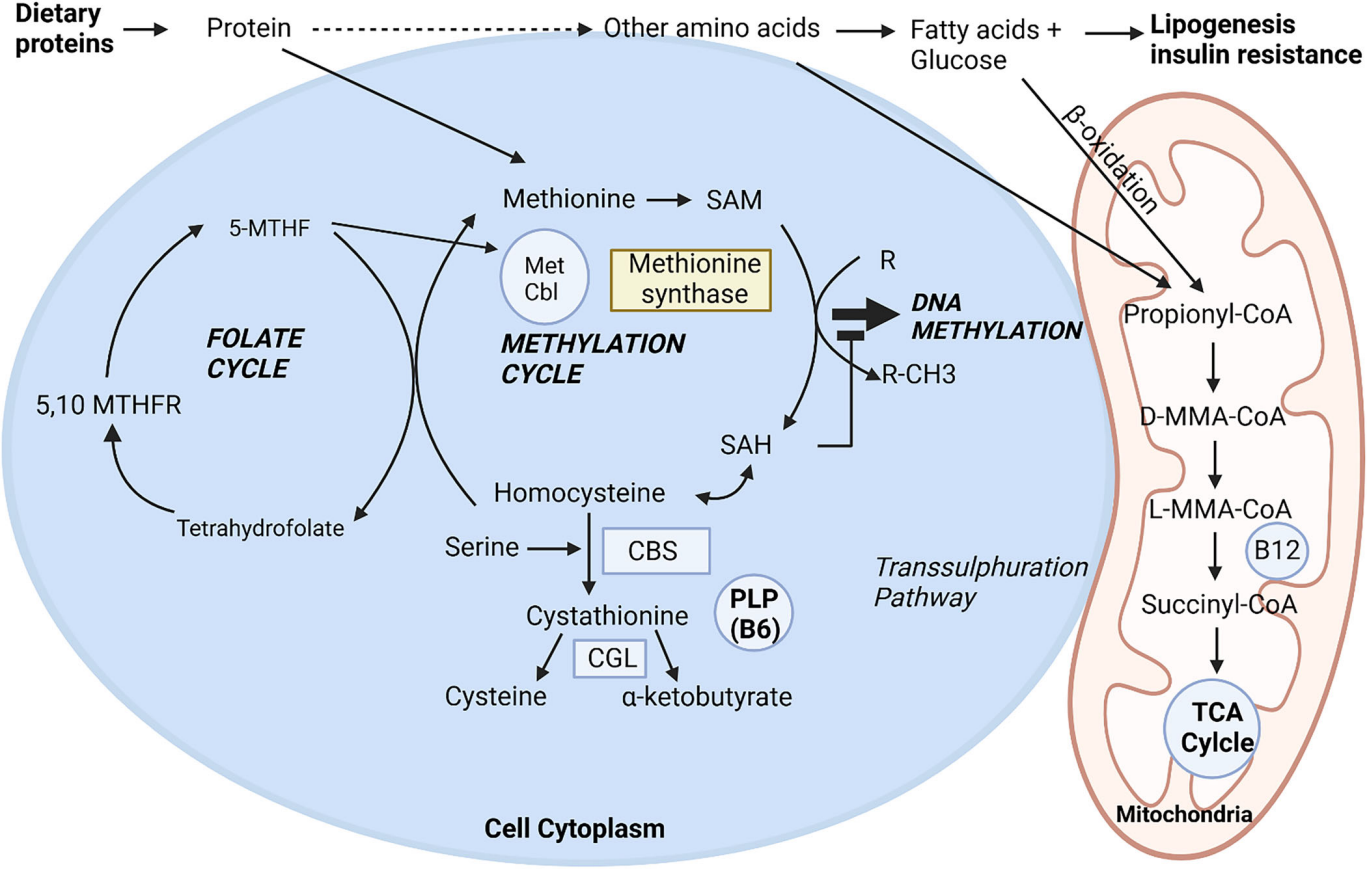
Metabolic pathway of cobalamin. Vitamin B12 is particularly involved in 2 metabolic pathways: (1) Homocysteine Metabolism Pathway. Vitamin B12, in the form of MetCbl, serves as a cofactor for methionine synthase, facilitating the conversion of homocysteine into methionine with the help of 5‐MTHF in the cell cytoplasm. Methionine subsequently forms SAM, a crucial compound involved in DNA methylation and neurotransmitter synthesis. SAM is then converted to SAH and hydrolysed back into homocysteine, completing the cycle. Additionally, in the presence of vitamin B6, homocysteine can be converted into cysteine, a precursor of the antioxidant glutathione, through alternative pathways. Moreover, the mitochondria play an indirect but significant role in the metabolism of methionine, homocysteine, and cysteine, primarily by reducing oxidative stress via the production of glutathione. When vitamin B12 or B6 levels are insufficient, homocysteine can accumulate, increasing ROS and resulting in oxidative damage. (2) Propionyl‐CoA Metabolism. Propionyl‐CoA, derived from the breakdown of amino acids such as valine, isoleucine, methionine and threonine, as well as from odd‐chain fatty acid oxidation, undergoes conversion to succinyl‐CoA. This multistep process involves: (I) carboxylation by propionyl‐CoA carboxylase with biotin (vitamin B7) as a cofactor, (II) isomerisation by methylmalonyl‐CoA racemase, and (III) a final conversion by methylmalonyl‐CoA mutase using adenosylcobalamin (vitamin B12) as a cofactor. Succinyl‐CoA subsequently enters the Krebs cycle (TCA Cycle), supporting ATP production and the synthesis of haem and neurotransmitters. Overall, a deficiency in vitamin B12 can lead to the accumulation of MMA, a biomarker that reflects impaired energy production and mitochondrial dysfunction. This disruption increases the risk of neuropathy due to energy metabolism defects in the Krebs cycle. Moreover, elevated MMA levels contribute to mitochondrial stress and cellular damage, underscoring the critical roles of vitamins B12 and B6 in energy production, antioxidant defence, and disease prevention. 5,10‐MTHFR, 5,10‐Methylenetetrahydrofolate reductase enzyme; 5‐MTHF, 5‐methyltetrahydrofolate; CBS, Cystathionine Beta Synthase enzyme; CGL, Cystathionine Gamma‐Lyase enzyme; D‐MMA‐CoA, D‐Methylmalonyl‐Coenzyme A; L‐MMA‐CoA, L‐Methylmalonyl‐Coenzyme A; MetCbl, methylcobalamin; MMA, methylmalonic acid; PLP, pyridoxal phosphate, the active form of vitamin B6 (pyridoxine); R, group bound to the nitrogenous base on which methylation occurs; RCH3, a structure in which a methyl group (–CH_3_) is attached to the rest of the molecule (R); ROS, reactive oxygen species; SAH, S‐adenosylhomocysteine; SAM, S‐adenosylmethionine; TCA Cycle, Krebs cycle.

Information on the absorption physiology and metabolic pathways of vitamin B12 is shown in Figures 3 and 4. Biomarkers employed to assess vitamin B12 status varied between studies. They included plasma vitamin B12, whole blood vitamin B12, plasma and urinary MMA, holoTC and plasma homocysteine.^42^ Vitamin B12 biomarker concentrations were assessed in four studies ^29^, ^30^, ^31^, ^33^ and three systematic reviews.^15^, ^39^, ^40^ Desmond et al. reported significantly lower vitamin B12 plasma concentrations in both unsupplemented and food‐fortified vegan groups, compared to omnivores. Vitamin B12 deficiency (defined as <148 pmol/L) was observed in 13% and low levels (148–258 pmol/L) in 40% of the vegan group.^30^ A significant difference in serum vitamin B12 levels, but not holoTC levels in vegan children compared to omnivores, was identified by Svetnicka et al.^33^ In this study, the majority, 59/69, that is 85.5% of vegan children, were supplemented, and only 2 vegan children were vitamin B12 deficient (defined as <156 μg/L).^33^ Alexy et al. found that 88% of vegan children were supplemented with B12, and only 8% had B12 deficiency. They defined biochemical vitamin B12 deficiency as holoTC < 35 pmol/L and MMA > 271 nmol/L.^29^ Finally, serum B12 concentration was adequate in a cohort of vegan Finnish children, with all but one child receiving supplements.^31^ Overall, vitamin B12 levels were lower in children and adolescents on vegan diets ^40^ and the prevalence of vitamin B12 deficiency was significantly higher in vegans (13% vs. 3.2% omnivorous).^39^

Svetnicka et al. also identified a significantly higher risk of developing high vitamin B12 levels in supplemented vegans, with plasma B12 levels >672 μg/L in 28/69 (40.5%) children.^33^ In their meta‐analysis, Neufingerl et al. reported that the majority of studies that assessed vitamin B12 status in supplemented vegans reported no significant differences compared to omnivores.^39^ Authors noted differences in the proportion of children on micronutrient supplements, as well as dosage and type of vitamins and minerals provided in the different studies.^33^, ^39^

#### 3.3.3 Vitamin D status

Three studies assessed 25(OH)‐vitaminD3 (25(OH)D) concentration.^29^, ^30^, ^31^ The prevalence of vitamin D supplementation varied between studies. Vitamin D levels were lower, and the prevalence of deficiency was greater in vegan children, even when given vitamin D supplements. Alexy et al. identified that 32% of children were 25(OH)D deficient, as 27% and 5% of vegan children had concentrations <20 and 12 ng/mL, respectively.^29^ Desmond et al. found that vegans who did not use supplements had lower 25(OH)D concentrations than omnivores.^30^ Interestingly, Hovinen et al.'s Finnish study reported significantly lower serum concentrations of 25(OH)D in vegans compared to omnivores, even though all vegans were supplemented and exposed to ample sunlight.^31^ It is important to mention that no information was available on the type of fortification or supplementation, whether of animal origin (D3 form) or plant‐source origin (D2 form), which is less effective at raising total 25(OH)D concentrations.^31^, ^43^

One study analysed bone mineral health, and reported a lower percentage of lumbar bone mineral content (delta difference in the fully adjusted model Δ −5.6 95% confidence interval [CI]: −10.6 to −0.5) and a lower percentage of total body bone mineral content (delta difference in the fully adjusted model Δ −3.7 95% CI: −7.0 to −0.4) in vegan children compared to omnivores (*p* < 0.05).^30^

#### 3.3.4 Other vitamins status

One study suggested that vegan participants might have a vitamin B2 deficiency (flavin adenine dinucleotide [FAD] < 199 μg/L).^29^ In another, serum concentrations of retinol‐binding protein (RBP) were used as a biomarker for vitamin A; RBP was found to be insufficient in all vegan children included (cut‐off 1.17 μmol/L).^31^

#### 3.3.5 Iron status

Three studies and two meta‐analyses assessed the impact of vegan diet on iron status and anaemia risk.^15^, ^29^, ^30^, ^31^, ^39^ Two studies reported a depleted iron status compared to omnivorous children and adolescents, with lower ferritin (μg/L), haemoglobin, and haematocrit.^29^, ^30^ Of note, the mean ferritin and haemoglobin concentrations were still within the normal range according to WHO criteria.^26^, ^27^ Two meta‐analyses found a lower mean ferritin value in vegans compared to omnivorous peers.^15^, ^39^ In addition, Desmond et al. found significantly more vegan children with mild anaemia (6% vegan vs. 0% omnivores), and depleted iron stores (30.2% of vegan vs. 12.8% omnivores) were detected.^30^ Neufingerl et al. concluded that the average prevalence of iron deficiency, or iron‐depleted stores, was higher in vegans, while the prevalence of anaemia was similar in vegans and omnivores.^39^

Iron supplementation and/or studies reporting it, as well as the criteria used to classify children, were heterogeneous.

#### 3.3.6 Iodine status

One study reported a significantly lower iodine concentration in spot urine (UIC)^38^ and significantly higher incidence of iodine deficiency (UIC < 100 μg/L) in vegan children compared to omnivores (41.9% vs. OM 19.6%, *p* = 0.06).^38^ The difference in UIC was not confirmed in another study.^31^

## 4 CONCLUSIONS

Nutritionally complete vegan diets that support normal growth and development in healthy infants, children and adolescents are potentially achievable but cannot be confirmed with the available evidence. Thus, vegan diets require close monitoring and supplementation with certain micronutrients, for example, iron, calcium, vitamin B12, and vitamin D. Expert dietary advice and supervision should be offered to parents.

Since the current vegan diet studies are primarily observational, the level of evidence is low. Furthermore, the biomarkers employed to assess nutritional status, particularly vitamin B12 status, varied across the studies, thus representing a limitation of the available literature. In addition, current publications have not reported whether children and their caregivers received professional nutritional counselling or dietary advice. Clinical research, well‐designed prospective studies, and high‐quality trials are required to address the current research gaps.

### Growth

- Vegan children (infants, toddlers, children and adolescents) had lower height as absolute value, but within the normal range for age, compared to omnivores. No difference was observed for height *z*‐score. Available literature is limited as it does not provide information whether the enroled subjects and/or their families received professional advice and guidance when adopting vegan diets.
- In the majority of studies conducted across all age groups (infants, toddlers, children, and adolescents) there was no significant difference in BMI *z*‐scores between vegans versus omnivores.
- Taking into account the quality of the studies and the heterogeneous information reported, evidence from the currently available publications is inconclusive.
- No substantial differences in growth patterns have been observed in vegan children compared to their vegetarian peers; however, the available evidence is still limited

### Nutritional intake

- Toddlers, children and adolescents following a vegan diet had a similar energy intake to omnivores.
- According to available literature, toddlers, children and adolescents following a vegan diet met the EFSA PRI for protein.
- Vegan toddlers, children and adolescents consumed more carbohydrates than omnivores, but intake was within EFSA Reference Intakes.
- Vegan children consumed more fibre than omnivores: toddlers and young children's intakes exceeded the EFSA AIs, whereas school‐age children and adolescent intakes were in line with EFSA recommendations.
- Compared to omnivores, vegan toddlers, children and adolescents had a consistently lower intake of SFAs and higher intake of PUFA, namely ALA and linoleic acid. Vegan children consumed higher‐quality dietary fats.
- Vegan toddlers, children and adolescents were at high risk of not meeting the EAR for vitamin B12 and at risk of not meeting the EAR for calcium
- In general, vegan toddlers, children and adolescents met the EAR for iron without supplementation.
- There is a lack of sufficient evidence to draw conclusions on other micronutrients.

### Laboratory biomarkers

The following conclusion on laboratory biomarkers refers to children and adolescents following a vegan diet without supplements:

- Vegan toddlers, children and adolescents had lower levels of total cholesterol, LDL cholesterol and HDL cholesterol compared to omnivores. For total cholesterol and LDL cholesterol, vegan children had acceptable values, but for HDL cholesterol, vegan children had borderline‐low levels according to reference values of the expert panel on integrated guidelines for cardiovascular health and risk reduction in children and adolescents.
- Vegan children from all age groups were at high risk of vitamin B12 deficiency.
- Vegan toddlers, children and adolescents were, like their omnivorous peers, at risk of vitamin D deficiency.
- Vegan children and adolescents had lower ferritin levels than omnivorous peers, even though mean ferritin values were within the normal range according to WHO criteria for age. Available literature indicates that vegan children and adolescents may be at risk of depleted iron stores.
- Vegan children and adolescents had lower haemoglobin levels than omnivores, although mean haemoglobin values were within the normal range according to WHO criteria for age. The available literature is inconclusive regarding the risk of anaemia.
- Available evidence is limited to comment on laboratory levels of other micronutrients.

## 5 RECOMMENDATIONS FOR ROUTINE CLINICAL PRACTICE

These recommendations are based on observational studies, systematic reviews and meta‐analyses that have been retrieved with a systematic search of the literature.

Taking into consideration the currently available evidence, ESPGHAN Nutrition Committee recommends:

### Growth

- The approach to monitoring growth and development in vegan children should be similar to the general paediatric population.

### Nutritional adequacy

- Children on a vegan diet should have regular nutritional counselling from a qualified paediatric dietitian. In settings where dietary support is not available, a public health approach should be adopted to provide clear advice based on evidence‐based research and clinical observation.
- Vegan diets in infants, children and adolescents should provide the same age‐appropriate average requirements for energy intake as for the general paediatric population.
- Vegan children may need to consume more protein than recommended for the general population in view of the lower digestibility of plant protein compared to animal protein.
- The protein intake should be assessed on an individual basis for each child. The quality as well as the quantity of protein should be taken into account to optimise essential amino acid intake.
- Foods rich in ALA should be consumed regularly to support adequate omega‐3 fatty acid ingestion. An algal‐based, age‐appropriate DHA supplement with titrated content might be considered in children aged <2 years.
- Nutritional advice should emphasise the intake of iron‐rich foods and nutrients such as vitamin C, which enhances iron absorption in view of the lower bioavailability of plant‐derived iron sources. Families should also be aware that nutrients such as phytates, polyphenols, calcium and oxalic acid inhibit iron absorption.
- Iron supplements should be considered in breastfed infants from 6 months and in older children if inadequate nutritional intake.
- To guarantee adequate calcium intake when introducing complementary feeding, breastfeeding and/or infant formula should be continued for 12 months or longer until the infant is on a nutritionally complete diet. Nutritional counselling should target calcium‐rich foods, e.g. calcium‐supplemented foods/fluids, and calcium supplements, where needed.
- Vegan children should aim to include vitamin B12 fortified foods in their diets, and a daily age‐appropriate vitamin B12 supplement is recommended (see Table S2).
- Consider an age‐appropriate vitamin D supplement (see Table S3) since a vegan diet is considered a risk factor for deficiency from birth to 18 years.^43^, ^44^
- In the same way as in the general population, Vitamin D supplements are even more important when there is a lack of skin (arms and face) exposure to sun, in more Northern countries, and/or an indoor lifestyle.
- If dietary calcium intake is insufficient, children benefit from a vitamin D and calcium supplement.

### Laboratory biomarkers

- Vitamin B12 metabolic parameters, such as plasma vitamin B12, plasma MMA and/or plasma homocysteine, should be monitored in all vegan children and adolescents during periods of rapid growth and/or suspicion of inadequate supplementation/intake.
- Other biochemical parameters, such as vitamin D and iron status, should be assessed if there is clinical suspicion of deficiency and/or insufficient intake.

## 6 PERSPECTIVES FOR FUTURE RESEARCH

Future research should encompass a variety of robust study designs, including clinical research evaluating educational programs, prospective longitudinal studies, and high‐quality trials, with clearly defined exposures and outcomes. This approach will help provide comprehensive and reliable evidence for the nutritional needs of infants and children adopting vegan diets. In view of the current available evidence, ESPGHAN Nutrition Committee recommends that future research in children on vegan diets should focus on.

### Growth

- High‐quality studies assessing growth parameters with a specific focus on length‐ or height‐for‐age SDSs.

### Nutritional intake

- Studies assessing calcium intake and associated bone mineral accretion.
- Studies reporting the prevalence and frequency of vegan children obtaining professional advice from a paediatric dietitian and monitoring by a paediatrician.

### Laboratory biomarkers

- High‐quality intervention studies to evaluate the risk of anaemia and iron depletion.
- High‐quality studies that evaluate the frequency and severity of vitamin B12 depletion, the usefulness of different Vitamin B12 biomarkers, and elucidate the most reliable biomarker to assess B12 deficiency in paediatric age groups.

## CONFLICT OF INTEREST STATEMENT

Elvira Verduci received honoraria, speaker's fee, congress fee, and/or participated in advisory boards of Nutricia, Nutricia Metabolics, and Nestlé. Jutta Kӧglmeier received a speaker's fee from Takeda. Nadja Haiden received honoraria, speaker's fee, congress fee, and/or participated in advisory boards of Baxter, Nutricia, Nestlé, HippMAM, and Medis. Laura Kivelä has received personal lecture fees from the Finnish Celiac Society and serves as a member of the advisory committee of the Finnish Celiac Society. Barbara de Koning received a speaker's fee from Fresenius‐Kabi. Susan Hill participated in advisory boards of Zealand, Vectiv‐Bio, and Takeda. Veronica Luque participated in industry‐funded research and/or consultancy for Nestlé, DGC, and BENEO. Sissel J. Moltu has received honoraria and speaker fees from Baxter, Nutricia, and NuMega. She has also received research funding (Formulaid™) from DSM Nutritional Products. Lorenzo Norsa received honoraria, speaker's fee, congress fee, and/or participated in advisory boards of Nestlè, Takeda, Zaeland, Danone, and Sanofi. Miguel Saenz De Pipaon received honoraria, speaker's fee, congress fee, and/or participated in advisory boards of Baxter, Nutrinia, Nutricia, Nestlé, and Fresenius. Francesco Savino has received a personal lecture fee from Nestlé, Biostime, and Junia Pharma. Jiri Bronsky received honoraria, speaker's fee, congress fee, and/or participated in advisory boards of AbbVie, MSD, Vitabalans, Nutricia, Nestlé, and Sanofi.

## Supporting information

Table S1. Search strategy of the systematic search according to different databases.

Table S2. Recommended intake of vitamin B12 in children on a vegan diet.

Table S3. Recommended vitamin D intake and supplementation in children on a vegan diet.

## References

[1] Tran E , Dale HF , Jensen C , et al. Effects of plant‐based diets on weight status: a systematic review. Diabetes Metab Syndr Obes. 2020;13:3433‐3448. 10.2147/DMSO.S272802 33061504 PMC7533223

[2] Rosenfeld DL , Burrow AL . Vegetarian on purpose: understanding the motivations of plant‐based dieters. Appetite. 2017;116:456‐463. 10.1016/j.appet.2017.05.039 28551111

[3] Turner‐McGrievy GM , Leach AM , Wilcox S , et al. Differences in environmental impact and food expenditures of four different plant‐based diets and an omnivorous diet: results of a randomized, controlled intervention. J Hunger Environ Nutr. 2016;11:382‐395. 10.1080/19320248.2015.1066734

[4] Springmann M , Wiebe K , Mason‐D'Croz D , et al. Health and nutritional aspects of sustainable diet strategies and their association with environmental impacts: a global modelling analysis with country‐level detail. Lancet Planet Health. 2018;2:e451‐e461. 10.1016/S2542-5196(18)30206-7 30318102 PMC6182055

[5] Borude S . Which is a good diet‐veg or non‐veg? Faith‐based vegetarianism for protection from obesity—a myth or actuality? Obes Surg. 2019;29:1276‐1280. 10.1007/s11695-018-03658-7 30604082

[6] Termannsen A‐D , Clemmensen KKB , Thomsen JM , et al. Effects of vegan diets on cardiometabolic health: a systematic review and meta‐analysis of randomized controlled trials. Obes Rev. 2022;23:e13462. 10.1111/obr.13462 35672940 PMC9540559

[7] Wang F , Zheng J , Yang B , et al. Effects of vegetarian diets on blood lipids: a systematic review and meta‐analysis of randomized controlled trials. J Am Heart Assoc. 2015;4:e002408. 10.1161/JAHA.115.002408 26508743 PMC4845138

[8] Alexy U , Fischer M , Weder S , et al. Food group intake of children and adolescents (6‐18 years) on a vegetarian, vegan or omnivore diet: results of the VeChi Youth Study. Br J Nutr. 2022;128:851‐862. 10.1017/S0007114521003603 34511141

[9] Hargreaves SM , Rosenfeld DL , Moreira AVB , et al. Plant‐based and vegetarian diets: an overview and definition of these dietary patterns. Eur J Nutr. 2023;62:1109‐1121. 10.1007/s00394-023-03086-z 36681744

[10] Euromonitor International . Where is the Vegan Claim Headed? Euromonitor International; 2021. https://www.euromonitor.com/article/where-is-the-vegan-claim-headed

[11] Desmond MA , Fewtrell MS , Wells JCK . Plant‐based diets in children: secular trends, health outcomes, and a roadmap for urgent practice recommendations and research. A systematic review. Nutrients. 2024;16:723. 10.3390/nu16050723 38474851 PMC10934552

[12] Simeone G , Bergamini M , Verga MC , et al. Do vegetarian diets provide adequate nutrient intake during complementary feeding? A systematic review. Nutrients. 2022;14:3591. 10.3390/nu14173591 36079848 PMC9459879

[13] Agnoli C , Baroni L , Bertini I , et al. Position paper on vegetarian diets from the working group of the Italian Society of Human Nutrition. Nutr Metab Cardiovasc Dis. 2017;27:1037‐1052. 10.1016/j.numecd.2017.10.020 29174030

[14] National Institute for Health and Care Excellence. Vitamin B12 Deficiency in Over 16s: Diagnosis and Management. NICE Guideline. 2024. 38713783

[15] Koller A , Rohrmann S , Wakolbinger M , et al. Health aspects of vegan diets among children and adolescents: a systematic review and meta‐analyses. Crit Rev Food Sci Nutr. 2023;64:13247‐13258. 10.1080/10408398.2023.2263574 37811643

[16] Amit M . Vegetarian diets in children and adolescents. Paediatr Child Health. 2010;15:303‐314. 21532796 PMC2912628

[17] Melina V , Craig W , Levin S . Position of the academy of nutrition and dietetics: vegetarian diets. J Acad Nutr Diet. 2016;116:1970‐1980. 10.1016/j.jand.2016.09.025 27886704

[18] Rudloff S , Bührer C , Jochum F , et al. Vegetarian diets in childhood and adolescence: position paper of the nutrition committee, German Society for Paediatric and Adolescent Medicine (DGKJ). Mol Cell Pediatr. 2019;6:4. 10.1186/s40348-019-0091-z 31722049 PMC6854160

[19] Redecilla Ferreiro S , Moráis López A , Moreno Villares JM , et al. Position paper on vegetarian diets in infants and children. Committee on Nutrition and Breastfeeding of the Spanish Paediatric Association]. An Pediatr (Engl Ed). 2020;92:306.e1‐306.e6. 10.1016/j.anpedi.2019.10.013 31866234

[20] Fewtrell M , Bronsky J , Campoy C , et al. Complementary feeding: a position paper by the European Society for Paediatric Gastroenterology, Hepatology, and Nutrition (ESPGHAN) Committee on Nutrition. J Pediatr Gastroenterol Nutr. 2017;64:119‐132. 10.1097/MPG.0000000000001454 28027215

[21] Villette C , Vasseur P , Lapidus N , et al. Vegetarian and vegan diets: beliefs and attitudes of general practitioners and pediatricians in France. Nutrients. 2022;14:3101. 10.3390/nu14153101 35956277 PMC9370229

[22] Moher D , Liberati A , Tetzlaff J , et al. Preferred reporting items for systematic reviews and meta‐analyses: the PRISMA statement. PLoS Med. 2009;6:e1000097. 10.1371/journal.pmed.1000097 19621072 PMC2707599

[23] Ouzzani M , Hammady H , Fedorowicz Z , et al. Rayyan—a web and mobile app for systematic reviews. Syst Rev. 2016;5:210. 10.1186/s13643-016-0384-4 27919275 PMC5139140

[24] European Food Safety Authority (EFSA). Dietary Reference Values for the EU. DRV Finder.

[25] Institute of Medicine. Dietary Reference Intakes: The Essential Guide to Nutrient Requirements. National Academies Press; 2006.

[26] World Health Organization (WHO) . Haemoglobin Concentrations for the Diagnosis of Anaemia and Assessment of Severity. Concentrations en hémoglobine permettant de diagnostiquer l'anémie et d'en évaluer la sévérité. World Health Organization (WHO); 2011.

[27] World Health Organization (WHO) . Serum Ferritin Concentrations for the Assessment of Iron Status and Iron Deficiency in Populations. Concentrations sériques de ferritine permettant d'évaluer le statut et les carences en fer dans les populations. World Health Organization (WHO); 2011.

[28] Expert Panel on Integrated Guidelines for Cardiovascular Health and Risk Reduction in Children and Adolescents; National Heart, Lung, and Blood Institute . Expert panel on integrated guidelines for cardiovascular health and risk reduction in children and adolescents: summary report. Pediatrics. 2011;128:S213‐S256. 10.1542/peds.2009-2107C 22084329 PMC4536582

[29] Alexy U , Fischer M , Weder S , et al. Nutrient intake and status of German children and adolescents consuming vegetarian, vegan or omnivore diets: results of the VeChi Youth Study. Nutrients. 2021;13:1707. 10.3390/nu13051707 34069944 PMC8157583

[30] Desmond MA , Sobiecki JG , Jaworski M , et al. Growth, body composition, and cardiovascular and nutritional risk of 5‐ to 10‐y‐old children consuming vegetarian, vegan, or omnivore diets. Am J Clin Nutr. 2021;113:1565‐1577. 10.1093/ajcn/nqaa445 33740036 PMC8176147

[31] Hovinen T , Korkalo L , Freese R , et al. Vegan diet in young children remodels metabolism and challenges the statuses of essential nutrients. EMBO Mol Med. 2021;13:e13492. 10.15252/emmm.202013492 33471422 PMC7863396

[32] Pandey S , Kashima S . Effects of dairy intake on anthropometric failure in children ages 6 to 23 mo consuming vegetarian diets and fulfilling minimum dietary diversity in India. Nutrition. 2021;91‐92:111446. 10.1016/j.nut.2021.111446 34587573

[33] Světnička M , Sigal A , Selinger E , et al. Cross‐sectional study of the prevalence of cobalamin deficiency and vitamin B12 supplementation habits among vegetarian and vegan children in the Czech Republic. Nutrients. 2022;14:535. 10.3390/nu14030535 35276893 PMC8838497

[34] Weder S , Hoffmann M , Becker K , et al. Energy, macronutrient intake, and anthropometrics of vegetarian, vegan, and omnivorous children (1–3 years) in Germany (VeChi Diet Study). Nutrients. 2019;11:832. 10.3390/nu11040832 31013738 PMC6521189

[35] Wirnitzer KC , Drenowatz C , Cocca A , et al. Health behaviors of Austrian secondary level pupils at a glance: first results of the from science 2 school study focusing on sports linked to mixed, vegetarian, and vegan diets. Int J Environ Res Public Health. 2021;18:12782. 10.3390/ijerph182312782 34886508 PMC8657632

[36] Weder S , Keller M , Fischer M , et al. Intake of micronutrients and fatty acids of vegetarian, vegan, and omnivorous children (1‐3 years) in Germany (VeChi Diet Study). Eur J Nutr. 2022;61:1507‐1520. 10.1007/s00394-021-02753-3 34855006 PMC8921058

[37] Weder S , Zerback EH , Wagener SM , et al. How does selenium intake differ among children (1‐3 years) on vegetarian, vegan, and omnivorous diets? Results of the VeChi Diet Study. Nutrients. 2022;15:34. 10.3390/nu15010034 36615692 PMC9824336

[38] Světnička M , Heniková M , Selinger E , et al. Prevalence of iodine deficiency among vegan compared to vegetarian and omnivore children in the Czech Republic: cross‐sectional study. Eur J Clin Nutr. 2023;77:1061‐1070. 10.1038/s41430-023-01312-9 37488261 PMC10630131

[39] Neufingerl N , Eilander A . Nutrient intake and status in children and adolescents consuming plant‐based diets compared to Meat‐Eaters: a systematic review. Nutrients. 2023;15:4341. 10.3390/nu15204341 37892416 PMC10609337

[40] Jensen CF . Vitamin B12 levels in children and adolescents on plant‐based diets: a systematic review and meta‐analysis. Nutr Rev. 2023;81:951‐966. 10.1093/nutrit/nuac096 36413044

[41] Hurrell R , Egli I . Iron bioavailability and dietary reference values. Am J Clin Nutr. 2010;91:1461S‐1467S. 10.3945/ajcn.2010.28674F 20200263

[42] Gerasimidis K , Bronsky J , Catchpole A , et al. Assessment and interpretation of vitamin and trace element status in sick children: a position paper from the European Society for Paediatric Gastroenterology Hepatology, and Nutrition Committee on Nutrition. J Pediatr Gastroenterol Nutr. 2020;70:873‐881. 10.1097/MPG.0000000000002688 32443051

[43] Devulapalli CS . Vitamin D intake and status in children and adolescents: comparing vegetarian, vegan, and omnivorous diets. Acta Paediatr. 2024;114:498‐504. 10.1111/apa.17463 39428613

[44] Saggese G , Vierucci F , Prodam F , et al. Vitamin D in pediatric age: consensus of the Italian Pediatric Society and the Italian Society of Preventive and Social Pediatrics, jointly with the Italian Federation of Pediatricians. Ital J Pediatr. 2018;44:51. 10.1186/s13052-018-0488-7 29739471 PMC5941617

