# Supplementary material for: Vegan diet and nutritional status in infants, children and adolescents: A position paper based on a systematic search by the ESPGHAN Nutrition Committee
Source: J Pediatr Gastroenterol Nutr. 2025 Aug 17;81(5):1318–45. doi: 10.1002/jpn3.70182 (PMC12580465; doi:10.1002/jpn3.70182)
Supplement: Supplementary file 1 — Table S1. Search strategy of the systematic search according to different databases. [file JPN3-81-1318-s001.docx]

**Supplemental Table S1**. Search strategy of the systematic review according to different databases.

| Database | Search Strategy | Filters applied | Articles Retrieved |
| --- | --- | --- | --- |
| MEDLINE/Pubmed | ((((((((((((((((((("vegan diet"[All Fields]) OR ("veg diet"[All Fields])) OR ("vegan consumers"[All Fields])) OR ("vegan dietary pattern"[All Fields])) OR ("vegan diet study"[All Fields])) OR ("vegan consumption"[All Fields])) OR ("vegan"[All Fields]))) OR ("vegetarian dietary pattern"[All Fields])) OR ("vegetarians"[MeSH Terms])) OR (diet, vegetarian[MeSH Terms])) OR (vegetarian diet[MeSH Terms])) OR ("diet, vegan/adverse effects"[MeSH Terms])) ) OR ("vegans"[MeSH Terms])) OR ("vegan diets"[All Fields])) OR ("vegetarian"[All Fields])) OR ("veganism"[All Fields])) OR ("vegetarianism"[All Fields]) AND (allchild[Filter])) AND ((((((((((((((((((((((((((((((((((((((("growth"[All Fields]) OR ("body growth"[All Fields])) OR ("catch up growth"[All Fields])) OR ("weight gain"[All Fields])) OR ("development"[All Fields])) OR ("adiposity"[All Fields])) OR ("obesity"[All Fields])) OR ("growth acceleration"[All Fields])) OR ("height"[All Fields])) OR ("bmi z score"[All Fields])) OR ("body mass"[All Fields])) OR ("weight status"[All Fields])) OR ("nutritional effect"[All Fields])) OR ("malnutrition"[All Fields])) OR ("nutritional state"[All Fields])) OR ("nutritional parameter"[All Fields])) OR ("body composition"[All Fields])) OR ("overweight"[All Fields])) OR ("weight excess"[All Fields])) OR ("nutritional risk"[All Fields])) OR ("micronutrient deficiency"[All Fields])) OR ("macronutrient deficiency"[All Fields])) OR ("iron deficiency"[All Fields])) OR ("zinc deficiency"[All Fields])) OR ("mineral deficiency"[All Fields])) OR ("vitamin deficiency"[All Fields])) OR ("trace element deficiency"[All Fields])) OR ("nutritional deficiency"[All Fields])) ) OR ("weight"[All Fields]) OR ("lenght"[All Fields]))))) OR ("nutrients"[All Fields])) OR ("macronutrient"[All Fields])) ) OR ("micronutrient"[All Fields])) OR ("nutritional intake"[All Fields])) OR ("nutritional status"[All Fields])) | Child: birth-18 years, from 2008 - 2023 | 411 |
| Embase | ('veg diet' OR 'vegan consumer' OR 'vegan dietary pattern' OR 'vegan diet study' OR 'vegan consumption' OR 'vegetarian'/exp/mj OR 'vegetarian diet'/exp/mj OR 'vegan diet'/exp/mj OR 'vegan'/exp/mj OR 'vegetarianism'/exp/mj OR 'vegetarian dietary pattern' OR 'vegan diets' OR 'veganism' OR 'veganism'/exp) AND ('growth'/exp OR 'growth' OR 'body growth'/exp OR 'body growth' OR 'catch up growth'/exp OR 'catch up growth' OR 'body weight gain'/exp OR 'body weight gain' OR 'development'/exp OR 'development' OR 'adiposity'/exp OR 'adiposity' OR 'obesity'/exp OR 'obesity' OR 'growth acceleration'/exp OR 'growth acceleration' OR 'height'/exp OR 'height' OR 'bmi z score'/exp OR 'bmi z score' OR 'body mass'/exp OR 'body mass' OR 'weight status'/exp OR 'weight status' OR 'nutritional effect' OR 'malnutrition'/exp OR 'malnutrition' OR 'nutritional state'/exp OR 'nutritional state' OR 'nutritional parameters'/exp OR 'nutritional parameters' OR 'body composition'/exp OR 'body composition' OR 'overweight'/exp OR 'overweight' OR 'weight excess' OR 'nutritional risk'/exp OR 'nutritional risk' OR 'nutritional deficiency'/exp OR 'nutritional deficiency' OR 'macronutrient deficiency' OR 'iron deficiency'/exp OR 'iron deficiency' OR 'zinc deficiency'/exp OR 'zinc deficiency' OR 'mineral deficiency'/exp OR 'mineral deficiency' OR 'vitamin deficiency'/exp OR 'vitamin deficiency' OR 'trace element deficiency'/exp OR 'trace element deficiency' OR 'weight'/exp OR 'weight' OR 'length'/exp OR 'length' OR 'nutrient'/exp OR 'nutrient' OR 'macronutrient'/exp OR 'macronutrient' OR 'micronutrient'/exp OR 'micronutrient' OR 'dietary intake'/exp OR 'dietary intake' OR 'nutritional status'/exp OR 'nutritional status') | ([adolescent]/lim OR [child]/lim OR [infant]/lim OR [preschool]/lim OR [school]/lim) AND [embase]/lim, from 2008 - 2023 | 287 |
| Cochrane CENTRAL | ((vegan diet):ti,ab,kw OR (veg diet):ti,ab,kw OR (vegan consumer):ti,ab,kw OR (vegan dietary pattern):ti,ab,kw OR (vegan diet study):ti,ab,kw (Word variations have been searched) OR (vegan consumption):ti,ab,kw OR (vegan):ti,ab,kw OR (vegetarian dietary pattern):ti,ab,kw OR (vegetarians):ti,ab,kw OR (vegetarian diet):ti,ab,kw OR (vegans):ti,ab,kw OR (vegan diets):ti,ab,kw OR (vegetarian):ti,ab,kw OR ("vegetarianism"):ti,ab,kw OR ("veganism"):ti,ab,kw) AND (growth OR body growth OR catch up growth OR body weight gain OR development OR adiposity OR obesity OR growth acceleration OR height OR bmi z score OR body mass OR weight status OR nutritional effect OR malnutrition OR nutritional state OR nutritional parameters OR body composition OR overweight OR weight excess OR nutritional risk OR nutritional deficiency OR macronutrient deficiency OR iron deficiency OR zinc deficiency OR mineral deficiency OR vitamin deficiency OR trace element deficiency OR weight OR length OR nutrient OR macronutrient OR micronutrient OR dietary intake OR nutritional status) | NOT ([embase]/lim AND [medline]/lim), from 2008 - 2023 | 280 |
