# Supplementary material for: Vegan diet and nutritional status in infants, children and adolescents: A position paper based on a systematic search by the ESPGHAN Nutrition Committee
Source: J Pediatr Gastroenterol Nutr. 2025 Aug 17;81(5):1318–45. doi: 10.1002/jpn3.70182 (PMC12580465; doi:10.1002/jpn3.70182)
Supplement: Supplementary file 2 — Table S2. Recommended intake of vitamin B12 in children on a vegan diet. [file JPN3-81-1318-s003.docx]

**Supplemental Table S2.** Recommended intake of vitamin B12 in children on a vegan diet.

| **Recommended intakes of vitamin B12 supplements in the population** | | | | |
| --- | --- | --- | --- | --- |
| **Age** | **EFSA* (AI)**  **(μg/day)** | **Multiple daily dose** | **Single daily dose **** | **Multiple weekly dose** |
| 6 - 12 months | 1.5 | 1 μg x2 | 5 μg | - |
| 1 - 3 years | 1.5 | 1 μg x2 | 5 μg | - |
| 4 - 6 years | 1.5 | 2 μg x2 | 25 μg | - |
| 7 – 10 years | 2.5 | 2 μg x2 | 25 μg | - |
| 11 -14 years | 3.5 | 2 μg x3 | 50 μg | 1000 μg x2 |
| 15 – 64 years | 4.0 | 2 μg x3 | 50 μg | 1000 μg x2 |
| 65 + years | 4.0 | 2 μg x3 | 50 μg | 1000 μg x2 |
| Pregnancy | 4.5 | 2 μg x3 | 50 μg | 1000 μg x2 |
| Breastfeeding | 5 | 2 μg x3 | 50 μg | 1000 μg x2 |

Modified from Agnoli, C.; et al. *Nutr Metab Cardiovasc Dis* **2017**, *27*, 1037–1052.

*European Food Safety Authority [European Food Safety Authority (EFSA)] Dietary Reference Values for the EU.

**The single daily dose compared to the multiple daily dose has higher intakes in consideration of the kinetics and absorption efficiency already described in the literature (Allen LH. *Am J Clin Nutr*. **2009**;89:693S-6S).

Abbreviations: Adequate Intake, AI.
