# Supplementary material for: Vegan diet and nutritional status in infants, children and adolescents: A position paper based on a systematic search by the ESPGHAN Nutrition Committee
Source: J Pediatr Gastroenterol Nutr. 2025 Aug 17;81(5):1318–45. doi: 10.1002/jpn3.70182 (PMC12580465; doi:10.1002/jpn3.70182)
Supplement: Supplementary file 3 — Table S3. Recommended vitamin D intake and supplementation in children on a vegan diet. [file JPN3-81-1318-s002.docx]

**Supplemental Table S3.** Recommended vitamin D intake and supplementation in children on a vegan diet.

| **Age** | **Adequate intake for general population (EFSA - AI)***  **(μg/day)** | **Supplementation for vegan diet**** | **Tolerable upper intake level (EFSA - UL)***  **(μg/day)** |
| --- | --- | --- | --- |
| 4-6 months | - | - Infants adopting vegan diet up to 1000 IU/day (25 (μg/day) **§** | 25  (1000 IU) |
| 7-11 months | 10  (400 IU) |  | 35  (1400 IU) |
| 1 - 3 years | 15  (600 IU) | - Children and adolescents adopting vegan diet up to 1000 IU/day (25 (μg/day) **§§** | 50  (2000 IU) |
| 4 - 6 years | 15  (600 IU) |  | 50  (2000 IU) |
| 7 – 10 years | 15  (600 IU) |  | 50  (2000 IU) |
| 11 -14 years | 15  (600 IU) |  | 100  (4000 IU) |
| 15 – 18 years | 15  (600 IU) |  | 100  (4000 IU) |

**§** According to Saggese et al., the risk factors for vitamin D deficiency during the first year of life are: Non-caucasian ethnicity with dark skin pigmentation; Vegan diet or inadequate diet; Chronic kidney disease; ; Hepatic failure and/or cholestasis; Malabsorption syndromes (i.e. cystic fibrosis, inflammatory bowel diseases, coeliac disease at diagnosis, etc.); Chronic therapies: anticonvulsants, systemic glucocorticoids, antiretroviral therapy, systemic antifungals (i.e. ketoconazole); Infants born from mothers with multiple risk factors for vitamin D deficiency, particularly in absence of vitamin D supplementation during pregnancy.

**§§** According to Saggese et al., the risk factors for vitamin D deficiency between 1 and 18 years are: Non-Caucasian ethnicity with dark skin pigmentation; Reduced sunlight exposure (due to lifestyle factors, chronic illness or hospitalization, complex disability, institutionalization, covering clothing for religious or cultural reasons) and/or constant use of sunscreens; International adoption; Obesity; Vegan diet or inadequate diet; Chronic kidney disease; Hepatic failure and/or cholestasis; Malabsorption syndromes (i.e. cystic fibrosis, inflammatory bowel diseases, coeliac disease at diagnosis, etc.); Chronic therapies: anticonvulsants, systemic glucocorticoids, antiretroviral therapy, systemic antifungals (i.e. ketoconazole).

*European Food Safety Authority [European Food Safety Authority (EFSA) Dietary Reference Values for the EU and European Food Safety Authority panel on dietetic products, nutrition, and allergies. Scientific opinion on dietary reference values for vitamin D. *EFSA J*. **2016**;14(10):4547.

**Saggese, G.; et al. *Ital J Pediatr* **2018**, *44*, 51.

Abbreviations: Adequate Intake, AI; Upper Level, UL; International Unit, IU.

Specification: 1 IU is equal to 0.025 µg of vitamin D
